# Effects of virtual reality and gamified digital rehabilitation on functional recovery in individuals with Spinal Cord Injury: a systematic review and Bayesian multilevel meta-analysis

**DOI:** 10.3389/fneur.2026.1954856

**Published:** 2026-10-01

**Authors:** Mengnan Xie, Mingyue Jiao, Zelong Duan, Jian Li, Yueying Zhu, Binbin Zhang, Fangfang Wen, Jifeng Long

**Affiliations:** 1 School of Physical Education, Shanxi University, Taiyuan, Shanxi, China; 2 Department of Physical Education, Lyuliang University, Lyuliang, Shanxi, China; 3 School of Teacher Education, Hezhou University, Hezhou, Guangxi, China; 4 Department of Physical Education, Shanxi Institute of Science and Technology, Jincheng, Shanxi, China; 5 Faculty of Sports and Leisure, Guangdong Ocean University, Zhanjiang, Guangdong, China

**Keywords:** Bayesian multilevel meta-analysis, functional recovery, gamified digital rehabilitation, Spinal Cord Independence Measure, Spinal Cord Injury, virtual reality

## Abstract

**Background:**

Virtual reality (VR) and gamified digital rehabilitation can increase task-specific practice and feedback for individuals with Spinal Cord Injury (SCI); however, their incremental effects on functional recovery remain uncertain.

**Methods:**

We searched PubMed, Web of Science, Embase, and the Cochrane Library from database inception through July 1, 2026, for randomized controlled trials (RCTs) evaluating VR or gamified digital rehabilitation in adults with SCI. Comparator conditions were classified as active rehabilitation, sham or attention control, or minimal/no intervention. The primary outcome was the Spinal Cord Independence Measure (SCIM). Hedges’ *g* estimates were synthesized using a Bayesian three-level random-effects model that accounted for within-study dependence. We estimated the 95% credible interval (CrI), 95% prediction interval (PI), and posterior probabilities of any benefit (*g* > 0) and of meeting a distribution-based threshold for clinical importance (*g* ≥ 0.40). A *post hoc* sensitivity analysis restricted the SCIM synthesis to trials with active-rehabilitation comparators. Seven secondary outcomes were synthesized separately. Risk of bias and certainty of evidence were assessed using the Cochrane Risk of Bias 2 tool (RoB 2) and the Grading of Recommendations Assessment, Development and Evaluation (GRADE) approach.

**Results:**

Thirteen RCTs involving 295 participants were included; comparator conditions were active rehabilitation in ten trials, sham or attention control in two, and no intervention in one. Seven studies contributed 25 eligible SCIM effect estimates. The pooled posterior mean effect favored digital rehabilitation but was small and imprecisely estimated (Hedges’ *g* = 0.24; 95% CrI, −0.12 to 0.61; 95% PI, −1.17 to 1.58). The posterior probabilities of any benefit and of a clinically important effect were 91.8 and 17.4%, respectively. Restricting the synthesis to the six SCIM studies with active-rehabilitation comparators yielded a similar, imprecise estimate (*g* = 0.28; 95% CrI, −0.20 to 0.75). Posterior mean effects for all seven secondary outcomes favored digital rehabilitation, although each 95% CrI included the null value. Direct posterior comparisons provided no clear evidence of effect modification by intervention category, injury completeness, or intervention duration. The weekly-dose association was uncertain and did not improve predictive performance. Overall risk of bias was rated as some concerns, and certainty was very low for all outcomes.

**Conclusion:**

VR and gamified digital rehabilitation may provide small functional benefits relative to the comparator conditions used in the included trials; however, clinically important and reproducible improvements have not been demonstrated. Current evidence is insufficient to prioritize specific technology types, patient subgroups, intervention durations, or weekly training doses.

**Systematic review registration:**

CRD420261458807; https://www.crd.york.ac.uk/PROSPERO/view/CRD420261458807

## 1 Introduction

Spinal Cord Injury (SCI) disrupts motor, sensory, and autonomic pathways, resulting in persistent limitations in self-care, mobility, and social participation. According to the Global Burden of Disease study, approximately 20.6 million people worldwide were living with SCI in 2019, with nearly 900,000 incident cases and approximately 6.2 million years lived with disability attributable to the condition (1). Although rehabilitation needs vary with neurological level, injury completeness, and stage of recovery, individuals with SCI highly value regaining function, reducing dependence on caregivers, and living more independently (2). Accordingly, the effectiveness of SCI rehabilitation should not be assessed solely using isolated measures such as muscle strength, walking speed, or performance on a specific task; it should also reflect whether improvements in these measures translate into clinically meaningful gains in independence in activities of daily living. The Spinal Cord Independence Measure (SCIM) was developed specifically for individuals with SCI and assesses self-care, respiration and sphincter management, and mobility. It has demonstrated good reliability, validity, and responsiveness to change and is therefore a core SCI-specific measure of functional independence (3).

Rehabilitation remains central to maximizing residual function, facilitating compensatory motor learning, and promoting activity-dependent neuroplasticity after SCI. Effective rehabilitation depends on repetitive, task-specific, and goal-directed practice, together with timely feedback on motor performance (4, 5). Virtual reality (VR) and gamified digital rehabilitation can incorporate therapeutic movements into interactive tasks and thereby increase practice repetition, task variety, and patient engagement through real-time visual or auditory feedback, adjustable difficulty, goal setting, scores, and rewards (4). Although related, VR rehabilitation and gamified digital rehabilitation are conceptually distinct. VR rehabilitation is defined primarily by interaction with a computer-generated environment and may be immersive, semi-immersive, or non-immersive, whereas gamified digital rehabilitation is defined by the use of game elements, such as goals, feedback, scores, and rewards, and does not necessarily require an immersive virtual environment. Maintaining this distinction may help disentangle effects related to virtual environments and levels of immersion from those related to gamification, rather than grouping all digital interventions into a single rehabilitation category. Clinical trials have evaluated VR and gamified digital rehabilitation for upper-limb and hand function, trunk control, sitting and standing balance, and gait, but their findings have been inconsistent. Early randomized controlled trials (RCTs) of platforms such as the Toyra system and Nintendo Wii reported good feasibility and acceptability; however, when added to conventional rehabilitation, these interventions did not consistently produce greater improvements in upper-limb function than dose-matched control training (6, 7). By contrast, some trials of gamified balance training or virtual soccer training reported between-group differences in sitting balance, dynamic balance, or gait performance (8, 9). These divergent findings may reflect differences in neurological level, injury completeness, time since injury, virtual environment, level of immersion, training task, intervention dose, and the intensity of control-group training. Importantly, improvement in a domain-specific motor outcome does not necessarily translate into greater overall functional independence. The SCIM total score is therefore an appropriate primary outcome for assessing the clinical value of digital rehabilitation, whereas measures of upper-limb function, muscle strength, and walking ability provide complementary evidence on specific dimensions of functional recovery.

Previous systematic reviews have examined gait and balance (10), overall functional performance (11), or upper-limb motor function (12), but have differed substantially in eligible study designs, participant characteristics, and outcome selection. A meta-analysis published in 2024 pooled data from five RCTs and four non-randomized studies; however, most outcome-specific analyses included only two studies, and no clear improvements were found in SCIM self-care, mobility, or total scores (11). Subsequent reviews were restricted to individuals with cervical SCI or incomplete SCI, limiting conclusions about overall effectiveness across neurological levels, degrees of injury completeness, and stages of rehabilitation (13, 14). A further methodological challenge is that a single RCT may contribute multiple intervention–control comparisons, SCIM effect estimates, or assessment time points. Because these estimates share participants, study settings, and intervention characteristics, they are statistically dependent. Selecting a single estimate discards potentially relevant information, whereas analyzing all estimates as independent observations can underestimate uncertainty. A three-level meta-analysis addresses this dependency by separately modeling sampling error, heterogeneity among effect sizes within studies, and heterogeneity between studies (15, 16). A Bayesian framework additionally permits direct probabilistic statements about the direction of treatment effects and the attainment of clinically important thresholds, while also providing prediction intervals (PIs) for the effects expected in future settings (17).

To address these limitations, we conducted a systematic review of RCTs involving adults with SCI, including both traumatic and non-traumatic etiologies and both complete and incomplete injuries, with the SCIM total score designated as the primary outcome. For SCIM outcomes, we calculated bias-corrected standardized mean differences (SMDs; Hedges’ *g*) using the Morris estimator for pretest–posttest–control-group designs and synthesized all eligible comparative estimates in a Bayesian three-level random-effects model that accounted for within-study dependence (16). The primary analysis estimated the overall posterior mean effect and its 95% credible interval (CrI) and 95% PI, together with the posterior probabilities of any benefit and of meeting a predefined distribution-based threshold for clinical importance. Additional analyses compared posterior effects between VR and gamified digital rehabilitation and across categories of injury completeness and intervention duration. We also explored the study-level association between weekly digital rehabilitation dose and SCIM effect estimates; this analysis was intended to examine an association rather than establish a causal dose–response relationship or identify an optimal treatment dose. Model robustness was examined using leave-one-study-out analyses and sensitivity analyses for prior specifications. Seven secondary outcomes—the Box and Block Test (BBT), upper-limb motor scores, grip strength, pinch strength, the 10-Meter Walk Test (10MWT), the Walking Index for Spinal Cord Injury (WISCI), and the Action Research Arm Test (ARAT)—were synthesized separately using outcome-specific Bayesian models and summarized in the main results table. These secondary outcomes were not subjected to the subgroup, clinical-threshold, dose–response, or extended sensitivity analyses reserved for the SCIM. By centering comprehensive statistical inference on functional independence while retaining supporting evidence from multiple functional domains, this study aimed to more fully characterize the overall effects, clinical importance, and potential effect modifiers of VR and gamified digital rehabilitation on functional recovery after SCI.

## 2 Methods and materials

### 2.1 Protocol and registration

This systematic review and Bayesian multilevel meta-analysis was conducted according to a prespecified protocol and reported in accordance with the Preferred Reporting Items for Systematic Reviews and Meta-Analyses (PRISMA) 2020 statement (18). The protocol was registered in the International Prospective Register of Systematic Reviews (PROSPERO; registration number CRD420261458807).

### 2.2 Eligibility criteria

Eligibility criteria were prespecified according to the participants, interventions, comparators, outcomes, and study design (PICOS) framework.

#### 2.2.1 Participants

Adults aged 18 years or older with traumatic or nontraumatic SCI were eligible, with no restrictions on neurological level, injury completeness, or rehabilitation phase.

#### 2.2.2 Interventions

Eligible interventions were required to include VR or gamified digital rehabilitation as a core therapeutic component, delivered either alone or as an adjunct to conventional rehabilitation. Interventions were classified into two categories:

VR or virtual environment rehabilitation. This category comprised interventions in which participants interacted with a computer-generated environment using immersive, semi-immersive, or non-immersive systems. Eligible examples included head-mounted displays, virtual walking or balance environments, avatar-based systems, the Gait Real-time Analysis Interactive Lab (GRAIL), and other systems in which therapeutic movements were performed within a virtual environment.

Gamified digital rehabilitation. This category comprised structured digital rehabilitation tasks that provided interactive feedback and incorporated at least one game-related element, such as explicit goals, scoring, progression, rewards, challenges, or immediate performance feedback. Eligible examples included Nintendo Wii, Xbox, Kinect, exergames, active video games, computer-assisted game training, robot-assisted gamified rehabilitation, MusicGlove, ReJoyce, and comparable digital platforms.

#### 2.2.3 Comparators

Eligible comparator conditions included usual care, physical therapy, occupational therapy, the same background rehabilitation without VR or gamification, sham or placebo versions of virtual interventions, attention-control interventions, other active rehabilitation interventions, wait-list controls, and no intervention. For reporting and interpretation, comparator conditions were classified into three categories: (1) active rehabilitation comparators, comprising usual care, physical or occupational therapy, dose-matched exercise without the eligible digital component, the same background rehabilitation, and other active rehabilitation interventions; (2) sham or attention-control comparators, which reproduced aspects of the intervention setting, equipment exposure, or therapist attention while omitting the intended therapeutic digital content; and (3) minimal-control comparators, comprising wait-list or no-intervention conditions. These categories were used to characterize the clinical contrast and were not treated as interchangeable.

#### 2.2.4 Outcomes

Studies were required to report at least one prespecified functional outcome at baseline and after the intervention and to provide sufficient data to calculate an effect estimate. When the reported information was insufficient, the relevant data had to be obtainable from the study authors.

The primary outcome was overall functional independence, assessed using the total score of an eligible version of the SCIM, including the original SCIM, the Spinal Cord Independence Measure III (SCIM-III), the Korean version of the Spinal Cord Independence Measure (K-SCIM), and the self-report Spinal Cord Independence Measure (SCIM-SR). Only SCIM total scores were classified as primary-outcome data.

The prespecified secondary outcomes were:

BBT;

Upper Extremity Motor Score (UEMS), including the American Spinal Injury Association Upper Extremity Motor Score (ASIA-UEMS);

grip strength;

pinch strength;

10MWT;

WISCI, including the Walking Index for Spinal Cord Injury II (WISCI-II); and

ARAT.

#### 2.2.5 Study design

Only RCTs were eligible.

### 2.3 Information sources and search strategy

We searched PubMed, Web of Science, Embase, and the Cochrane Library from database inception through July 1, 2026. The search was restricted to English-language publications.

The search strategies combined database-specific controlled vocabulary, including Medical Subject Headings (MeSH) and Emtree terms, with free-text terms covering three concepts: SCI, VR or gamified digital rehabilitation, and RCTs. The PubMed search strategy was as follows:

(“Spinal Cord Injuries”[Mesh] OR “Injuries, Spinal Cord”[tiab] OR “Cord Injuries, Spinal”[tiab] OR “Cord Injury, Spinal”[tiab] OR “Injury, Spinal Cord”[tiab] OR “Spinal Cord Injury”[tiab] OR “Spinal Cord Injuries”[tiab] OR “Traumatic Myelopathy”[tiab] OR “Traumatic Myelopathies”[tiab] OR “Spinal Cord Trauma”[tiab] OR “Spinal Cord Traumas”[tiab] OR “Post-Traumatic Myelopathy”[tiab] OR “Post Traumatic Myelopathy”[tiab] OR “Spinal Cord Contusion”[tiab] OR “Spinal Cord Contusions”[tiab] OR “Spinal Cord Laceration”[tiab] OR “Spinal Cord Lacerations”[tiab] OR “Spinal Cord Transection”[tiab] OR “Spinal Cord Transections”[tiab]) AND (“Robotics”[Mesh] OR robot*[tiab] OR “robot-assisted”[tiab] OR “robotic-assisted”[tiab] OR “robot assisted”[tiab] OR “robotic assisted”[tiab] OR “Telemedicine”[Mesh] OR “Smartphone”[Mesh] OR “Mobile Applications”[Mesh] OR “Medical Informatics”[Mesh] OR tele*[tiab] OR mobile*[tiab] OR app*[tiab] OR phone*[tiab] OR mHealth[tiab] OR smartphone*[tiab] OR cellphone*[tiab] OR tablet*[tiab] OR computer*[tiab] OR eHealth[tiab] OR message*[tiab] OR internet*[tiab] OR video*[tiab] OR videoconf*[tiab] OR “personal digital assistant”[tiab] OR “medical informatics”[tiab] OR exergaming[tiab] OR gaming[tiab] OR game*[tiab] OR “exer-gaming”[tiab] OR “digital exercise”[tiab] OR “active videogame”[tiab] OR Xbox[tiab] OR Wii[tiab] OR “Wii Fit”[tiab] OR Kinect[tiab] OR “virtual game”[tiab] OR “virtual gaming”[tiab] OR “Virtual Reality”[Mesh] OR “Augmented Reality”[Mesh] OR “virtual reality”[tiab] OR “augmented reality”[tiab] OR “mixed reality”[tiab] OR “smart glasses”[tiab] OR virtual*[tiab] OR digital*[tiab] OR immersive*[tiab] OR “computer-assisted”[tiab] OR “computer assisted”[tiab] OR “Biofeedback, Psychology”[Mesh] OR “Neurofeedback”[Mesh] OR biofeedback*[tiab] OR neurofeedback*[tiab] OR feedback*[tiab] OR wearable*[tiab] OR acceleromet*[tiab] OR gyroscop*[tiab] OR sensor*[tiab] OR shoe*[tiab] OR insole*[tiab] OR “Electric Stimulation Therapy”[Mesh] OR “physical stimulation”[tiab] OR “peripheral stimulation”[tiab]) AND random*.

The full search strategies for all databases are provided in Supplementary File 1.

### 2.4 Study selection

All retrieved records were imported into EndNote X9. Duplicate records were removed using automated matching followed by manual verification.

Two reviewers independently screened the titles and abstracts of all records. Any record judged potentially eligible by either reviewer was retrieved for full-text assessment. The same reviewers then independently assessed the full-text reports against the prespecified eligibility criteria and documented a specific reason for each exclusion. Disagreements were resolved through discussion and re-examination of the source reports; unresolved disagreements were adjudicated by a third reviewer.

### 2.5 Data collection

Two reviewers independently extracted data using a standardized, pilot-tested extraction form developed for this review. The independently extracted data were compared, and discrepancies were resolved by consensus after re-examination of the source reports. Any unresolved discrepancies were adjudicated by a third reviewer.

Data were obtained from primary publications, Supplementary materials, trial protocols, registry records, and related reports. Multiple reports from the same trial were linked and consolidated into a single study record. Data were reconciled across reports, with priority given to the most complete source and to directly reported numerical values. Separate outcome-specific datasets were created for the SCIM, BBT, UEMS, grip strength, pinch strength, 10MWT, WISCI, and ARAT.

The extracted data included publication details, the country of the research institution, funding information, study design, and sample size; participant age and sex or gender distribution, as reported; SCI etiology, neurological level, injury completeness, American Spinal Injury Association Impairment Scale (AIS) grade, rehabilitation phase, and time since injury; descriptions of the intervention and comparator; comparator category; background rehabilitation and other co-interventions received by each group; whether treatment duration or therapist contact time was planned to be matched, when reported; devices or platforms used; intervention category and level of immersion; mode of supervision and rehabilitation setting; intervention duration, number of sessions per week, duration of each eligible digital rehabilitation session, and total number of sessions; outcome assessment time points; and group-specific sample sizes, means, and standard deviations at baseline and postintervention.

Only training time attributable to eligible VR or gamified components was counted as digital rehabilitation exposure. Weekly digital rehabilitation dose was calculated as follows:


$$\begin{array}{l} \text{Weekly digital rehabilitation dose}\;(\min/\text{week})\\ =\text{Number of sessions}\;\mathrm{per}\;\text{week}\times \text{Duration of each}\\ \;\;\;\;\;\;\;\;\;\text{digital rehabilitation session}\;(\min) \end{array}$$

Cumulative digital rehabilitation dose was calculated as follows:


$$\begin{array}{l} \text{Cumulative digital rehabilitation dose}\;(\min)\\ =\text{Weekly digital rehabilitation dose}\;(\min/\text{week})\\ \times \text{Intervention duration}\;(\text{weeks}) \end{array}$$

When individual participant data were available in Supplementary materials, the required summary statistics were calculated directly from the participants included in the reported analysis. When numerical results were reported only graphically, values were extracted using WebPlotDigitizer version 4.1 (19), and a second reviewer independently verified the extracted values against the source figures. When outcome data were missing, unclear, or internally inconsistent, the corresponding authors were contacted for clarification or additional information.

### 2.6 Risk-of-bias assessment

Risk of bias was assessed using the Cochrane Risk of bias 2 tool for randomized trials (RoB 2) (20). The tool evaluates five domains: bias arising from the randomization process, bias due to deviations from intended interventions, bias due to missing outcome data, bias in measurement of the outcome, and bias in selection of the reported result. Based on the domain-specific signaling questions and the RoB 2 algorithms, domain-level and overall judgments were categorized as low risk of bias, some concerns, or high risk of bias.

Two reviewers independently completed the assessments. Disagreements were resolved through discussion and re-examination of the source reports; unresolved disagreements were adjudicated by a third reviewer. Risk-of-bias judgments informed the assessment of the certainty of evidence. For the primary SCIM outcome, we prespecified a risk-of-bias sensitivity analysis conditional on the availability of study subsets with meaningfully different risk-of-bias profiles.

### 2.7 Certainty-of-evidence assessment

The Grading of Recommendations Assessment, Development and Evaluation (GRADE) approach was used to assess the certainty of evidence for the primary SCIM outcome and the seven secondary outcomes (21). Because all included studies were RCTs, the body of evidence for each outcome was initially rated as high certainty. Certainty was downgraded by one or two levels, as appropriate, for serious or very serious concerns regarding risk of bias, inconsistency, indirectness, or imprecision, or for strong or very strong suspicion of publication bias. Final certainty was classified as high, moderate, low, or very low.

For the SCIM, the assessment considered the distribution of RoB 2 judgments; the pooled posterior mean Hedges’ *g* and its 95% CrI and 95% PI; heterogeneity between studies and among effect sizes within studies; and the posterior probabilities of any benefit and of meeting the prespecified distribution-based threshold for clinical importance. For the secondary outcomes, GRADE judgments considered the pooled effect estimate, 95% CrI, between-study heterogeneity, risk-of-bias profile, directness of the evidence, and the amount of outcome-specific information available. Bayesian estimates informed, but did not mechanically determine, the GRADE judgments.

Two reviewers independently assessed each GRADE domain and documented the rationale for any downgrading. Disagreements were resolved through discussion, with adjudication by a third reviewer when necessary. Outcome-specific certainty ratings and reasons for downgrading are presented in the Summary of Findings tables.

### 2.8 Effect-size calculation

All prespecified functional outcomes were continuous. Effect estimates were calculated as SMDs and reported as Hedges’ *g*. Within each outcome domain, standardization permitted the synthesis of results obtained using different instrument versions, score ranges, measurement units, or assessment formats. Hedges’ small-sample correction was applied to reduce bias in studies with small sample sizes.

For the primary SCIM outcome, effect sizes were calculated using the pretest–post test–control-group estimator described by Morris (22). For each eligible intervention–control comparison, the between-group difference in mean change from baseline to post intervention was standardized by the pooled baseline standard deviation:


$$g=J\times \frac{\left({\bar{X}}_{\text{post},I}-{\bar{X}}_{\mathrm{pre},I})-({\bar{X}}_{\text{post},C}-{\bar{X}}_{\mathrm{pre},C}\right)}{S_{\mathrm{pre},\text{pooled}}}$$

In this equation, *I* and *C* denote the intervention and control groups, respectively, and *J* denotes the exact small-sample correction factor for Hedges’ *g*, calculated using *n_I_* + *n_C_* − 2 degrees of freedom. The pooled baseline standard deviation was calculated as follows:


$$S_{\mathrm{pre},\text{pooled}}=\sqrt{\frac{(n_{I}-1)S_{\mathrm{pre},I}^{2}+(n_{C}-1)S_{\mathrm{pre},C}^{2}}{n_{I}+n_{C}-2}}$$

When the number of participants included in the analysis differed between baseline and postintervention because of loss to follow-up, the smaller sample size was used for the corresponding study group. The sampling variance of Hedges’ *g* was estimated as follows:


$$v_{g}=2(1-r)(\frac{1}{n_{I}}+\frac{1}{n_{C}})+\frac{g^{2}}{2(n_{I}+n_{C})}$$

In these equations, *r* denotes the within-participant correlation between baseline and postintervention measurements. Because primary studies rarely reported this correlation, a value of *r* = 0.50 was assumed for the primary analyses, consistent with recommended practice for synthesizing change-score data when empirical estimates are unavailable (23). The assumed value affects the estimated sampling variance but not the point estimate of Hedges’ *g*.

Effect directions were harmonized so that positive values consistently favored VR or gamified digital rehabilitation. Higher scores indicate better function for the SCIM, BBT, UEMS, grip strength, pinch strength, WISCI, and ARAT. For the 10MWT, when the outcome was reported as time to test completion, the effect estimate was multiplied by −1 because shorter completion times indicate better walking performance.

For the SCIM, a distribution-based range of clinically important effects was defined as 0.40–0.50 pooled baseline standard deviations (24). Hedges’ *g* = 0.40 was prespecified as the primary threshold, whereas *g* = 0.50 was retained as an upper reference value. The primary threshold was used to estimate the posterior probability that the pooled SCIM effect met or exceeded a clinically important magnitude. No clinical-importance threshold was applied to the seven secondary outcomes.

Secondary outcomes were synthesized using direction-harmonized Hedges’ *g* estimates. Their 95% CrIs and between-study random-effects standard deviations were reported in the outcome-specific pairwise meta-analysis tables. Analyses reserved for the SCIM—including clinical-threshold probabilities, subgroup comparisons, weekly-dose meta-regression, PIs, leave-one-study-out analyses, and extended sensitivity analyses of prior specifications and model assumptions—were not conducted for the secondary outcomes. As summarized in Table 1, all eight outcomes were expressed on the SMD scale, and the direction of 10MWT completion-time effects was reversed so that positive values consistently favored digital rehabilitation.

**Table 1:** Pairwise meta-analysis for all outcomes.

Outcomes	SMD	Lower	Upper	PSRF	SD (intercept)	SD-lower	SD-upper	GRADE	Bulk_ESS	Tail_ESS
		95% CrI	95% CrI			95% CrI	95% CrI			
10MWT	0.25	−0.57	1.01	1.00	0.48	0.02	1.58	Very low^a^^,^^b^^,^^c^	9,933	10,364
ARAT	0.16	−0.53	0.78	1.00	0.37	0.01	1.34	Very low^a^^,^^b^^,^^c^	11,522	9,817
BBT	0.24	−0.58	0.99	1.00	0.42	0.01	1.46	Very low^a^^,^^b^^,^^c^	13,101	11,609
Grip strength	0.16	−0.35	0.63	1.00	0.30	0.01	1.02	Very low^a^^,^^b^^,^^c^	14,211	12,003
Pinch strength	0.14	−0.45	0.74	1.00	0.51	0.06	1.34	Very low^a^^,^^b^^,^^c^	11,164	13,056
UEMS	0.19	−0.45	0.79	1.00	0.32	0.01	1.09	Very low^a^^,^^b^^,^^c^	12,983	11,471
WISCI	0.59	−0.80	1.79	1.00	1.26	0.26	2.93	Very low^a^^,^^b^^,^^c^	11,206	13,166
SCIM	0.24	−0.12	0.61	1.00	0.21	0.01	0.69	Very low^a^^,^^b^^,^^c^	10,343	11,709

Bulk_ESS (Bulk Effective Sample Size) indicates the effective sample size of the Markov chain Monte Carlo samples in the main distribution region. Tail_ESS evaluates the effective sample size in the tail region. Because lower 10MWT times indicate better walking performance, the original effect direction was multiplied by −1 so that positive SMD values favour the intervention. Higher Action Research Arm Test scores indicate better upper-extremity independence, and higher WISCI scores indicate better walking independence; therefore, no direction reversal was applied for these outcomes. 10MWT, 10-metre walk test; ARAT, Action Research Arm Test; BBT, Box and Block Test; CrI, credible interval; ESS, effective sample size; PSRF, potential scale reduction factor; SD, standard deviation; SMD, standardized mean difference; UEMS, Upper Extremity Motor Score; WISCI, Walking Index for Spinal Cord Injury.

a Risk of bias.

b Inconsistency.

c Imprecision.

### 2.9 Synthesis methods

A study was included in an outcome-specific synthesis if it reported sufficient baseline and postintervention data to calculate direction-harmonized Hedges’ *g* estimates for that outcome. Each eligible intervention–control comparison was retained as a separate effect estimate. When a study contributed more than one eligible SCIM effect estimate, each estimate was assigned a unique effect-level identifier, while all estimates from the same study shared a common study-level identifier. This hierarchical data structure allowed all eligible SCIM effect estimates to be retained and their within-study dependence to be modeled.

The comprehensive synthesis framework described below was applied only to the SCIM. The seven secondary outcomes—the BBT, UEMS, grip strength, pinch strength, 10MWT, WISCI, and ARAT—were synthesized separately using outcome-specific Bayesian pairwise random-effects meta-analyses. Effect estimates were not pooled across outcome domains. For each secondary outcome, Table 1 reports the posterior pooled Hedges’ *g* and its 95% CrI, the between-study random-effects standard deviation and its 95% CrI, the certainty-of-evidence rating, the potential scale reduction factor, and the bulk and tail effective sample sizes. Analyses reserved for the SCIM—including clinical-threshold probabilities, PIs, subgroup comparisons, weekly-dose meta-regression, leave-one-study-out analyses, and extended sensitivity analyses of prior specifications—were not conducted for the secondary outcomes.

The prespecified primary SCIM model included all eligible control conditions. Because each treatment effect was defined relative to its corresponding control condition, the pooled estimate was interpreted as the average relative effect across the control conditions represented in the eligible trials. We did not conduct a formal moderator analysis by control type because the SCIM evidence base included only one study with a minimal-control condition and no studies with sham or attention-control conditions; thus, any between-category estimate would have been determined by a single study outside the active-control category. We therefore conducted a *post hoc* sensitivity analysis restricted to SCIM studies with active-rehabilitation controls, using the same three-level model and prespecified analytical approach as in the primary analysis. Control-type-stratified analyses were not conducted for the secondary outcomes because each outcome was informed by only three or four studies. Instead, the distribution of control types was reported descriptively and considered when interpreting the pooled estimates.

#### 2.9.1 Primary SCIM model

The primary SCIM synthesis was conducted using a Bayesian three-level random-effects meta-analytic model to account for dependence among multiple effect estimates nested within studies (16, 17). Let $y_{\mathrm{ij}}$ denote the observed Hedges’ *g* estimate for effect i in study j, and let $s_{\mathrm{ij}}$ denote its sampling standard error (SE), which was treated as known. The model was specified as follows:


$$y_{\mathrm{ij}}|\theta_{\mathrm{ij}}∼\text{Normal}(\theta_{\mathrm{ij}},s_{\mathrm{ij}}^{2}),$$

$$\theta_{\mathrm{ij}}=\mu +u_{j}+v_{\mathrm{ij}},$$

$$u_{j}∼\text{Normal}(0,\tau_{\text{study}}^{2}),v_{\mathrm{ij}}∼\text{Normal}(0,\tau_{\text{effect}}^{2})$$

In this model, *μ* denotes the overall pooled effect for the SCIM outcome, $u_{j}$ denotes the study-level random deviation for study *j*, and $v_{\mathrm{ij}}$ denotes the effect-level random deviation for effect *i* within study *j*. The standard deviations of $u_{j}$ and $v_{\mathrm{ij}}$ quantify between-study and within-study heterogeneity, respectively. Accordingly, level 1 represents the known sampling variance of each Hedges’ *g* estimate, level 2 captures residual heterogeneity among effect estimates within studies beyond sampling error, and level 3 captures heterogeneity between studies. In brms, the SEs were supplied as known quantities using the se() term, and the observation-level residual standard deviation was fixed at zero. Thus, all variability beyond the known sampling variance was represented by the study-level and effect-level random effects, with no additional residual-error term.

The model assumed a Gaussian likelihood. Weakly informative priors were assigned to all estimated parameters on the SMD scale. A normal prior was assigned to the overall pooled effect:


μ∼Normal(0,1),

Half-Student-*t* priors were assigned to the between-study and within-study heterogeneity standard deviations:


$$\tau_{\text{study}},\tau_{\text{effect}}∼\text{half}-\text{Student}-t(3,0,1).$$

The prior for the overall pooled effect was centered at zero but allowed effects of clinically important magnitude, whereas the half-Student-*t* priors concentrated probability density near zero heterogeneity while retaining heavy right tails to accommodate substantial heterogeneity when supported by the data (25).

The primary model was fitted using eight Markov chain Monte Carlo (MCMC) chains, each run for 6,000 iterations, including 3,000 warm-up iterations, yielding 24,000 post-warm-up draws in total. To reduce divergent transitions and improve exploration of the posterior distribution, the Hamiltonian Monte Carlo tuning parameters adapt_delta and max_treedepth were set to 0.99 and 15, respectively.

#### 2.9.2 Posterior summary and heterogeneity

The overall treatment effect is summarized by the posterior mean of *μ* and its 95% equal-tailed CrI. Positive values favor VR-based or gamified digital rehabilitation with respect to functional independence. In addition to the interval estimate, the posterior probability of a beneficial treatment effect is calculated as follows:


Pr(μ>0),

and the posterior probability that the overall effect exceeds the prespecified distribution-based threshold for clinical importance:


Pr(μ≥0.40).

A value of *g* = 0.40 is adopted as the primary threshold, representing the lower bound of the prespecified clinically important range and corresponding to 0.4 times the pooled baseline standard deviation; *g* = 0.50 is retained as the upper-bound reference value (24). Evidence of benefit and clinical importance is evaluated using these posterior probabilities rather than frequentist *p* values.

Between-study and effect-level heterogeneity are characterized by the posterior distributions of $\tau_{\text{study}}$ and $\tau_{\text{effect}}$ , respectively. Because the posterior distributions of heterogeneity parameters may be right-skewed, heterogeneity is summarized primarily by the posterior median and 95% CrI.

A 95% PI is constructed for the true effect expected in a new study. For each posterior draw, new study-level and effect-level deviations are independently sampled from their respective distributions, conditional on the corresponding heterogeneity parameters:


$$\theta_{\mathrm{new}}=\mu +u_{\mathrm{new}}+v_{\mathrm{new}},$$

where


$$u_{\mathrm{new}}∼\text{Normal}(0,\tau_{\text{study}}^{2}),$$

and


$$v_{\mathrm{new}}∼\text{Normal}(0,\tau_{\text{effect}}^{2}).$$

The 2.5th and 97.5th percentiles of the posterior predictive draws define the A 95% PI. This interval quantifies uncertainty in the true effect expected in a new study, incorporating heterogeneity at both the study and effect levels. It does not, however, include the additional sampling error associated with the effect estimate that would be observed in that study.

For the graphical presentation, a partially pooled posterior effect is estimated for each study. Although all eligible effect estimates contribute to model estimation, the displayed study-level estimate retains the study-specific random intercept while excluding the individual effect-level deviations. It therefore represents the underlying mean SMD for that study rather than any individual observed comparison. The posterior distributions of the study-specific and overall effects are displayed in a combined forest and ridgeline density plot. Study-specific estimates are presented with 95% CrIs, whereas the overall estimate is additionally accompanied by the 95% PI and the distribution-based thresholds for clinical importance.

#### 2.9.3 Subgroup analyses

Exploratory Bayesian subgroup analyses were conducted for three study-level moderators specified *a priori*:

1. *Intervention category:* gamified digital rehabilitation versus rehabilitation based on VR or virtual environments;
2. *Injury completeness:* mixed samples comprising participants with complete SCI and those with incomplete SCI versus samples restricted to participants with incomplete SCI; and
3. *Intervention duration:* ≤ 4 weeks versus > 4 weeks.

Each moderator was coded at the study level according to the relevant study and intervention characteristics and assigned to all eligible SCIM effect estimates contributed by that study. For each moderator, a no-intercept Bayesian three-level meta-regression model was fitted:


$$y_{\mathrm{ij}}|s_{\mathrm{ij}}∼0+\text{subgroup}+(1|\text{study})+(1|\text{effect}).$$

The no-intercept parameterization enabled direct estimation of the subgroup-specific mean effects. Normal priors were assigned to the subgroup-specific coefficients:


$$\beta_{k}∼\text{Normal}(0,1).$$

Half-Student-*t*3,0,1 priors were assigned to the standard deviations of the study- and effect-level random effects. The subgroup models used the same MCMC settings as the primary model: 8 chains, 6,000 iterations per chain, including 3,000 warm-up iterations, adapt_delta = 0.99, and a maximum tree depth of 15.

For each subgroup, the posterior mean SMD, 95% CrI, and posterior probability of benefit, Pr(SMD>o) , were reported. Effect modification was evaluated using the posterior distribution of the direct between-subgroup contrast, defined as the effect in the second subgroup minus that in the first. The contrast was summarized by its posterior mean, 95% CrI, and the posterior probabilities that it was above or below zero. A subgroup difference was not inferred merely because the 95% CrI excluded zero for one subgroup but included zero for the other; instead, inference was based on the posterior distribution of the direct contrast. Results for subgroups represented by fewer than two independent studies were interpreted descriptively.

#### 2.9.4 Exploratory meta-regression of weekly digital rehabilitation dose

An exploratory Bayesian multilevel meta-regression was conducted to examine the association between the study-level weekly dose of digital rehabilitation and the SCIM effect size. Weekly dose was defined as the total number of minutes of eligible digital rehabilitation delivered per week. If weekly dose was not reported directly, it was calculated as follows:


$$\begin{array}{l} \text{Weekly dose}=\text{sessions}\;\mathrm{per}\;\text{week}\times \text{digital rehabilitation}\\ \;\;\;\;\;\;\;\;\;\;\;\;\;\;\;\;\;\;\;\;\;\;\;\;\;\;\;\;\;\;\;\;\;\;\;\;\text{minutes}\;\mathrm{per}\;\text{session}. \end{array}$$

Only eligible digital rehabilitation time delivered to the intervention group was counted. When a study reported multiple valid weekly-dose values, their study-level mean was used. Studies with missing, nonfinite, or nonpositive weekly-dose values were excluded, and the meta-regression was performed only when at least four independent studies provided valid dose data.

Weekly dose was $\log_{2}$ -transformed so that the regression coefficient represented the mean change in Hedges’ g associated with a doubling of weekly dose. The transformed moderator was centered at the mean of the $\log_{2}$ -transformed doses across independent studies, with each study contributing once regardless of the number of eligible effect estimates it provided:


$$x_{j}=\log_{2}(D_{j})-\frac{1}{J}\sum_{k=1}^{J}\log_{2}(D_{k})$$

where $D_{j}$ denotes the weekly dose in study j , and J denotes the number of independent studies included in the meta-regression.

The dose model was specified in mixed-effects formula notation as follows:


$$y_{\mathrm{ij}}|s_{\mathrm{ij}}∼\mu +\beta_{\text{dose}}x_{j}+(1|\text{study})+(1|\text{effect}),$$

where $\beta_{\text{dose}}$ represents the mean change in SCIM Hedges’ *g* associated with a doubling of the weekly digital rehabilitation dose. A Normal(0,1) prior was assigned to the intercept, a weakly informative Normal(0,0.5) prior was assigned to the dose coefficient, and half-Student- t(3,0,1) priors were assigned to the study- and effect-level random-effect standard deviations.

An intercept-only random-effects model that omitted the weekly-dose term was refitted to the same complete-case dataset and served as the null model. Both models were estimated using 8 chains, with 6,000 iterations per chain, including 3,000 warm-up iterations, adapt_delta = 0.99, a maximum tree depth of 15. The posterior distribution of $\beta_{\text{dose}}$ was summarized by its mean, median, 95% CrI, $\mathrm{Pr}(\beta_{\text{dose}}>0)$ , and $\mathrm{Pr}(\beta_{\text{dose}}<0)$ .

The predictive performance of the dose and null models was compared using Pareto-smoothed importance-sampling leave-one-out cross-validation (PSIS-LOO) (26). Model comparison was summarized using the expected log predictive density under leave-one-out cross-validation ( $\mathrm{ELP}D_{\mathrm{LOO}}$ ), its SE, the leave-one-out information criterion (LOOIC), and the corresponding between-model differences. Changes in $\tau_{\text{study}}$ and $\tau_{\text{effect}}$ after inclusion of the weekly-dose moderator were also examined.

Posterior predictions were generated only over the observed range of weekly doses. Study-level inverse-variance-weighted SMDs were displayed solely as descriptive points in the dose–effect plots; all inferential analyses retained the eligible effect-level estimates and their multilevel structure. Because weekly dose was measured at the aggregate study level, the findings were interpreted as exploratory ecological associations. They were not considered evidence of a causal dose–response relationship and were not used to identify an optimal therapeutic dose, threshold, or inflection point.

#### 2.9.5 Sensitivity and influence analyses

The influence of individual studies on the primary pooled SCIM effect was assessed using a leave-one-study-out analysis, in which each study was excluded in turn and the primary Bayesian multilevel model was refitted. After each exclusion, the pooled SMD, its 95% CrI, Pr(SMD>0) , and posterior summaries of $\tau_{\text{study}}$ and $\tau_{\text{effect}}$ were re-estimated. Changes relative to the full-data model were recorded, including whether the 95% CrI changed from including zero to excluding zero, or vice versa. These influence models were estimated using 4 chains, with 5,000 iterations per chain, including 2,500 warm-up iterations, adapt_delta = 0.99, and a maximum tree depth of 15. A parallel leave-one-study-out analysis of the weekly-dose coefficient, $\beta_{\text{dose}}$ , was conducted using 4 chains, with 4,000 iterations per chain, including 2,000 warm-up iterations.

Sensitivity to prior specification was assessed by refitting the primary model under three alternative heterogeneity-prior specifications. In all models, the prior for the overall pooled effect, μ , was held constant at Normal(0,1) . The alternative priors for the heterogeneity parameters were specified as follows:

##### 2.9.5.1 Conservative specification


$$\begin{array}{l} \tau_{\text{study}}∼\text{Half}-\text{Normal}(0,0.20),\\ \tau_{\text{effect}}∼\text{Half}-\text{Student}-t(3,0,0.50). \end{array}$$

##### 2.9.5.2 Wider study-level half-normal specification


$$\begin{array}{l} \tau_{\text{study}}∼\text{Half}-\text{Normal}(0,0.50),\\ \tau_{\text{effect}}∼\text{Half}-\text{Student}-t(3,0,0.50). \end{array}$$

##### 2.9.5.3 Half-Student-*t* specification


$$\begin{array}{l} \tau_{\text{study}}∼\text{Half}-\text{Student}-t(3,0,0.50),\\ \tau_{\text{effect}}∼\text{Half}-\text{Student}-t(3,0,0.50). \end{array}$$

Each prior-sensitivity model was estimated using 4 chains, with 5,000 iterations per chain, including 2,500 warm-up iterations. Sensitivity to prior specification was evaluated by comparing the pooled SMD, its 95% CrI, Pr(SMD>0) , and the posterior distributions of the two heterogeneity parameters across models.

The planned risk-of-bias sensitivity analysis considered three datasets: (1) all studies; (2) studies remaining after the exclusion of those judged to be at high overall risk of bias; and (3) studies judged to be at low risk of bias or to raise some concerns. These models were specified using broad half-normal priors for the heterogeneity parameters. An analysis was considered estimable only when the full dataset included at least one study at high risk of bias and at least three independent studies remained after the exclusion of all such studies. When the risk-of-bias assessments did not yield distinct analytical subsets, the corresponding analysis was reported as “not estimable” rather than repeating results for identical datasets.

#### 2.9.6 Computation and model diagnostics

All Bayesian models were fitted in R version 4.5.1 using the *brms* package, which provides an interface to Stan for Bayesian multilevel modeling (27). Posterior summaries were obtained using the *posterior* package, and PSIS-LOO model comparisons were performed using the *loo* package (28). Forest plots, posterior ridgeline density plots, dose–effect plots, and diagnostic plots were produced using *ggplot2*, *ggridges*, and related R packages.

Convergence and sampling efficiency were assessed using trace plots across chains, the rank-normalized split potential scale reduction factor ( $\hat{R}$ ), bulk and tail effective sample sizes, and the number of divergent transitions (29). Posterior predictive checks were conducted by comparing the distribution of the observed SCIM effect estimates with that of replicated estimates generated from the posterior predictive distribution of the primary fitted model, using density-overlay plots. Posterior probabilities, CrIs, and PIs were interpreted directly within the Bayesian framework.

## 3 Results

### 3.1 Study selection

The databases were searched from inception through July 2026. The search identified 10,104 records. After 2,067 duplicates were removed, 8,037 unique records underwent title and abstract screening. Of these, 56 full-text articles were assessed for eligibility, and 13 (7, 9, 30–40) studies met the prespecified eligibility criteria and were ultimately included. The study-selection process is shown in Figure 1.

**Figure 1:**
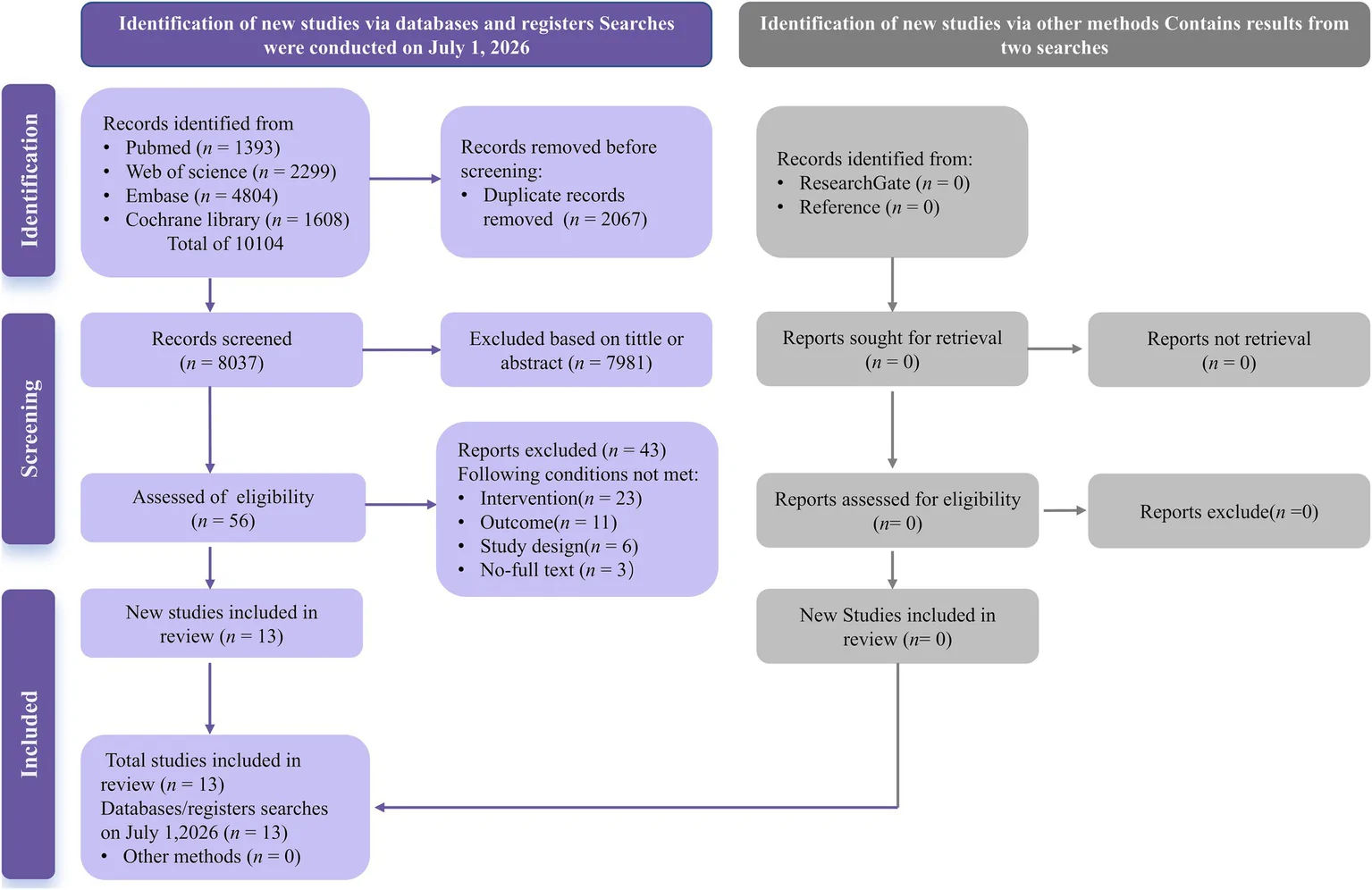
PRISMA flow diagram depicting the study selection.

### 3.2 Study characteristics

The 13 included RCTs enrolled 295 participants and were published between 2011 and 2026. Ten studies (76.9%) used active rehabilitation comparators, two (15.4%) used sham or attention controls, and one (7.7%) used a no-intervention comparator. Among the seven studies contributing SCIM data, six used active rehabilitation comparators and one used a no-intervention comparator; none used a sham or attention control. Detailed descriptions and classifications of the comparators are provided in Supplementary Section 2.1. The trials were conducted in seven countries; South Korea (4/13, 30.8%), Spain (3/13, 23.1%), and India (2/13, 15.4%) were the most frequently represented. Eight studies (61.5%) reported receiving research funding, four (30.8%) reported no funding, and one (7.7%) did not report its funding status.

#### 3.2.1 Study populations

The study populations were clinically heterogeneous with respect to neurological level, injury completeness, and rehabilitation phase. Eight studies enrolled participants with cervical SCI, two enrolled participants with thoracic or thoracolumbar SCI, two enrolled samples with mixed injury levels (cervical, thoracic, or lumbar), and one did not report the neurological level of injury. Seven studies enrolled only participants with incomplete SCI, five enrolled participants with both complete and incomplete SCI, and one enrolled only participants with complete SCI. With respect to rehabilitation phase, seven studies focused on chronic SCI, four on acute or subacute SCI, and two included participants across multiple phases. Neurological impairment ranged from AIS grades A to D, and both tetraplegia and paraplegia were represented.

#### 3.2.2 Interventions

Seven studies (53.8%) evaluated rehabilitation based on VR or virtual environments, whereas six (46.2%) evaluated gamified digital rehabilitation. Nine interventions (69.2%) were non-immersive, and four (30.8%) were fully immersive. Technologies included head-mounted VR systems, Nintendo Wii, virtual walking environments, MusicGlove, ReJoyce, Armeo Power or Armeo Spring, Amadeo, Diego, Pablo, and robotic gait training combined with immersive VR and brain–computer interface support. Therapeutic tasks primarily targeted upper-limb and hand function, trunk control, lower-limb movement, balance, or walking ability. Three interventions were delivered primarily in home settings, whereas the remainder were delivered in inpatient, outpatient, hospital, rehabilitation-center, or laboratory settings. Intervention duration ranged from 3 to 36 weeks (median, 5 weeks), training frequency ranged from 3 to 5 sessions per week (median, 3 sessions), and reported digital rehabilitation time ranged from 10 to 72.5 min per session (median, 30 min).

#### 3.2.3 Outcome measures

The SCIM was the most commonly reported outcome and was assessed in seven studies (53.8%). UEMS, grip strength, and pinch strength were each reported in four studies (30.8%), whereas the BBT, 10MWT, WISCI, and ARAT were each reported in three studies (23.1%). Several trials reported multiple functional outcomes, and some contributed more than one eligible comparison or assessment time point. Detailed information on study design, participants, injury characteristics, interventions, and outcomes is provided in Supplementary Files 2, 4.

### 3.3 Risk of bias within studies

The results of the RoB 2 assessments are presented in Supplementary File 3. All 13 studies received an overall risk-of-bias judgment of “some concerns.” The most frequent concerns involved bias due to deviations from intended interventions and bias in selection of the reported result, with nine studies (9/13, 69.2%) rated as having “some concerns” in each domain. Seven studies (53.8%) were rated as having “some concerns” for bias arising from the randomization process. Risk-of-bias judgments were more favorable for bias due to missing outcome data and bias in measurement of the outcome: in each domain, seven studies (53.8%) were rated as low risk and six (46.2%) as having “some concerns.”

### 3.4 Certainty of evidence

The certainty of evidence was rated as very low for all eight evaluated outcomes: SCIM, BBT, UEMS, grip strength, pinch strength, 10MWT, WISCI, and ARAT (Table 1).

### 3.5 Meta-analysis results

#### 3.5.1 Primary outcome: SCIM

Seven studies involving 164 participants contributed 25 eligible SCIM effect estimates to the Bayesian three-level meta-analysis. The pooled posterior mean SMD was 0.24 (95% CrI, −0.12 to 0.61), with positive values favoring VR-based or gamified digital rehabilitation over the comparator conditions. The posterior probability that the pooled effect was greater than zero was 91.8%. In contrast, the posterior probability that the effect exceeded the prespecified distribution-based threshold for clinical importance (Hedges’ *g* = 0.40) was 17.4%. The 95% PI for the true effect of a new eligible comparison in a new study ranged from −1.17 to 1.58, indicating substantial uncertainty and allowing for effects favoring either the intervention or the comparator (Figures 2, 3).

**Figure 2:**
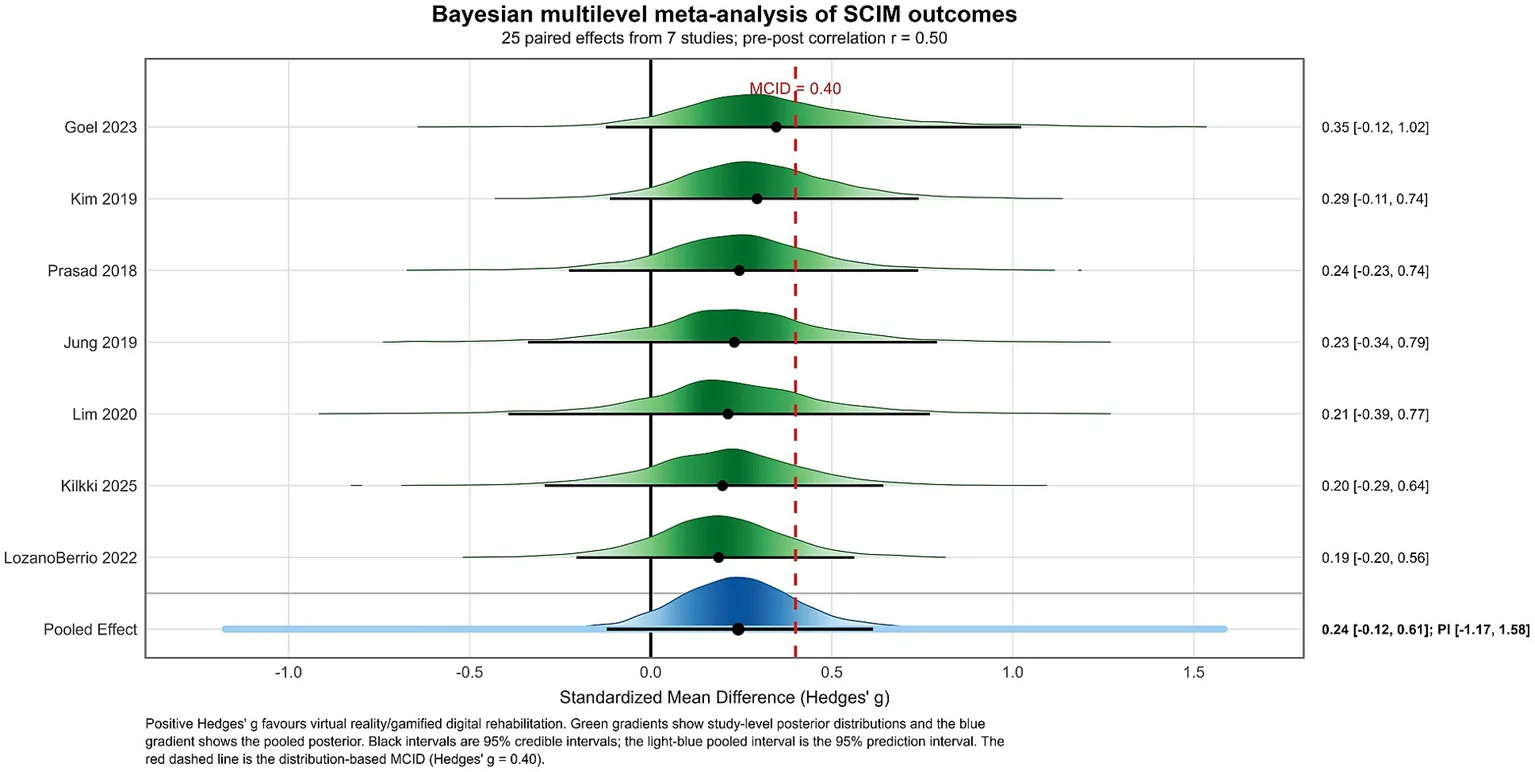
Bayesian multilevel meta-analysis of SCIM outcomes. Positive Hedges’ *g* favours virtual reality/gamified digital rehabilitation. Green gradients show study-level posterior distributions, and the blue gradient shows the pooled posterior distribution. Black intervals represent 95% credible intervals, whereas the light-blue pooled interval represents the 95% prediction interval. The red dashed line indicates the distribution-based minimal clinically important difference (MCID; Hedges’ *g* = 0.40).

**Figure 3:**
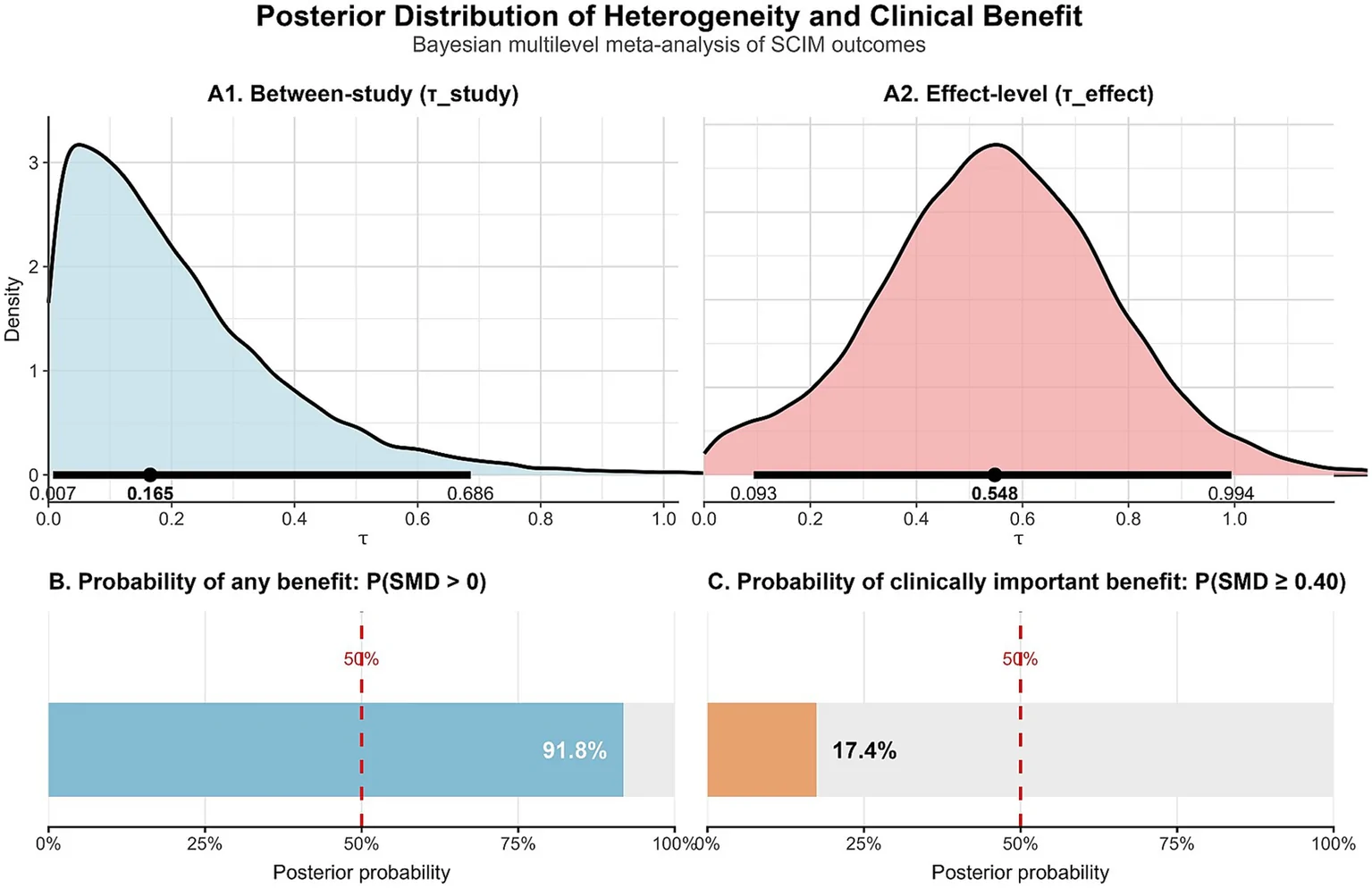
Posterior distributions of heterogeneity and clinical benefit. **(A1)** shows between-study heterogeneity and **(A2)** shows effect-level heterogeneity. **(B)** shows the posterior probability that the pooled SCIM effect is greater than zero. **(C)** shows the posterior probability that the pooled effect reaches the distribution-based MCID of 0.40 Hedges’ *g*. These posterior probabilities are not frequentist *p* values.

The partially pooled study-specific posterior mean SMDs were all positive and ranged from 0.19 to 0.35, with corresponding posterior probabilities of benefit ranging from 82.3 to 93.1%. However, all study-specific 95% CrIs included zero, and the posterior probabilities of exceeding the distribution-based threshold for clinical importance ranged from 11.9 to 35.9%. The study by Goel et al. (40) had the largest study-specific posterior mean (SMD, 0.35; 95% CrI, −0.12 to 1.02), whereas the study by Lozano-Berrio et al. (34) had the lowest posterior probability of exceeding the threshold (11.9%).

The posterior median of between-study heterogeneity, $\tau_{\text{study}}$ , was 0.17 (95% CrI, 0.01 to 0.69), compared with 0.55 (95% CrI, 0.09 to 0.99) for effect-level heterogeneity, $\tau_{\text{effect}}$ . The higher posterior median for $\tau_{\text{effect}}$ suggested greater variation among eligible effects within studies than between independent studies, although both heterogeneity estimates remained imprecise (Figure 3). Overall, most of the posterior mass for the pooled SCIM effect lay above zero, favoring the intervention, but the magnitude of the average effect remained uncertain. The posterior probability of exceeding the prespecified threshold for clinical importance was low, and the wide PI indicated that the true effect in a new setting could vary substantially in both magnitude and direction.

#### 3.5.2 Secondary functional outcomes

The posterior mean SMDs for all seven secondary outcomes favored VR-based or gamified digital rehabilitation, but the 95% CrIs for all outcomes included zero (Table 1). Among the walking-related outcomes, the pooled posterior mean SMD was 0.59 (95% CrI, −0.80 to 1.79) for WISCI and 0.25 (95% CrI, −0.57 to 1.01) for the 10MWT. WISCI had the largest posterior mean effect among the secondary outcomes but also the widest CrI and the greatest estimated between-study heterogeneity ( $\tau_{\text{study}=1.26}$ ; 95% CrI, 0.26 to 2.93).

For upper-limb function and muscle strength, the pooled posterior mean SMDs were 0.24 (95% CrI, −0.58 to 0.99) for BBT, 0.19 (95% CrI, −0.45 to 0.79) for UEMS, 0.16 (95% CrI, −0.53 to 0.78) for ARAT, 0.16 (95% CrI, −0.35 to 0.63) for grip strength, and 0.14 (95% CrI, −0.45 to 0.74) for pinch strength. Estimated between-study heterogeneity for these outcomes ranged from 0.30 for grip strength to 0.51 for pinch strength. Accordingly, the secondary-outcome estimates remained too imprecise to support clear conclusions regarding improvements in specific domains of motor function, muscle strength, manual dexterity, or walking ability.

### 3.6 Subgroup analyses

The results of the SCIM subgroup analyses are presented in Supplementary File 5 and were interpreted using direct posterior contrasts between subgroups.

For intervention category, four studies of gamified digital rehabilitation contributed 18 effect estimates, with a posterior mean SMD of 0.18 (95% CrI, −0.30 to 0.64; Pr[SMD>0] = 80.8%). Three studies of rehabilitation based on VR or virtual environments contributed seven effect estimates, with a posterior mean SMD of 0.36 [95% CrI, −0.31 to 1.02; Pr(SMD>0) = 87.4%]. The direct contrast, defined as the VR/virtual-environment subgroup minus the gamified digital rehabilitation subgroup, had a posterior mean of 0.19 (95% CrI, −0.63 to 0.98). The posterior probability that the effect was larger in the VR/virtual-environment subgroup was 69.1%. The broad posterior distribution of the contrast did not provide clear evidence that intervention category modified the SCIM effect.

For injury completeness, studies enrolling mixed samples of participants with complete and incomplete SCI had a posterior mean SMD of 0.21 [four studies and 17 effect estimates; 95% CrI, −0.29 to 0.69; Pr(SMD>0) = 83.8%]. Studies restricted to participants with incomplete SCI had a posterior mean SMD of 0.28 [three studies and eight effect estimates; 95% CrI, −0.38 to 0.92; Pr(SMD>0) = 81.7%]. The direct contrast, defined as the incomplete-only subgroup minus the mixed subgroup, was 0.06 (95% CrI, −0.75 to 0.88), with a 56.6% posterior probability that the effect was larger in the incomplete-only subgroup. These results did not provide clear evidence that the SCIM effect differed according to injury completeness.

For intervention duration, regimens lasting ≤4 weeks had a posterior mean SMD of 0.39 [four studies and 12 effect estimates; 95% CrI, −0.14 to 0.88; Pr(SMD>0)=93.8%] , whereas regimens lasting >4 weeks had a posterior mean SMD of 0.09 [three studies and 13 effect estimates; 95% CrI, −0.44 to 0.61; Pr(SMD>0)=65.2% ]. The direct contrast, defined as >4 weeks minus ≤4 weeks, was −0.30 (95% CrI, −1.03 to 0.44). The posterior probability that the effect was smaller for regimens lasting >4 weeks was 82.0%. Although the posterior distribution placed greater probability on a larger effect for interventions lasting ≤4 weeks, the contrast remained too imprecise to identify intervention duration as an effect modifier.

### 3.7 Exploratory weekly-dose meta-regression

All seven studies contributing SCIM data, comprising 25 eligible effect estimates, were included in the exploratory study-level meta-regression of weekly digital rehabilitation dose. The posterior mean dose coefficient, $\beta_{\text{dose}}$ , was 0.27 (95% CrI, −0.46 to 0.97), corresponding to an average increase of 0.27 in Hedges’ g for each doubling of weekly dose. The posterior probabilities that the coefficient was greater than and less than zero were 78.0 and 22.0%, respectively (Figure 4, Supplementary File 6). Thus, the posterior distribution placed more probability on a positive association but remained compatible with both negative associations and positive associations of substantial magnitude.

**Figure 4:**
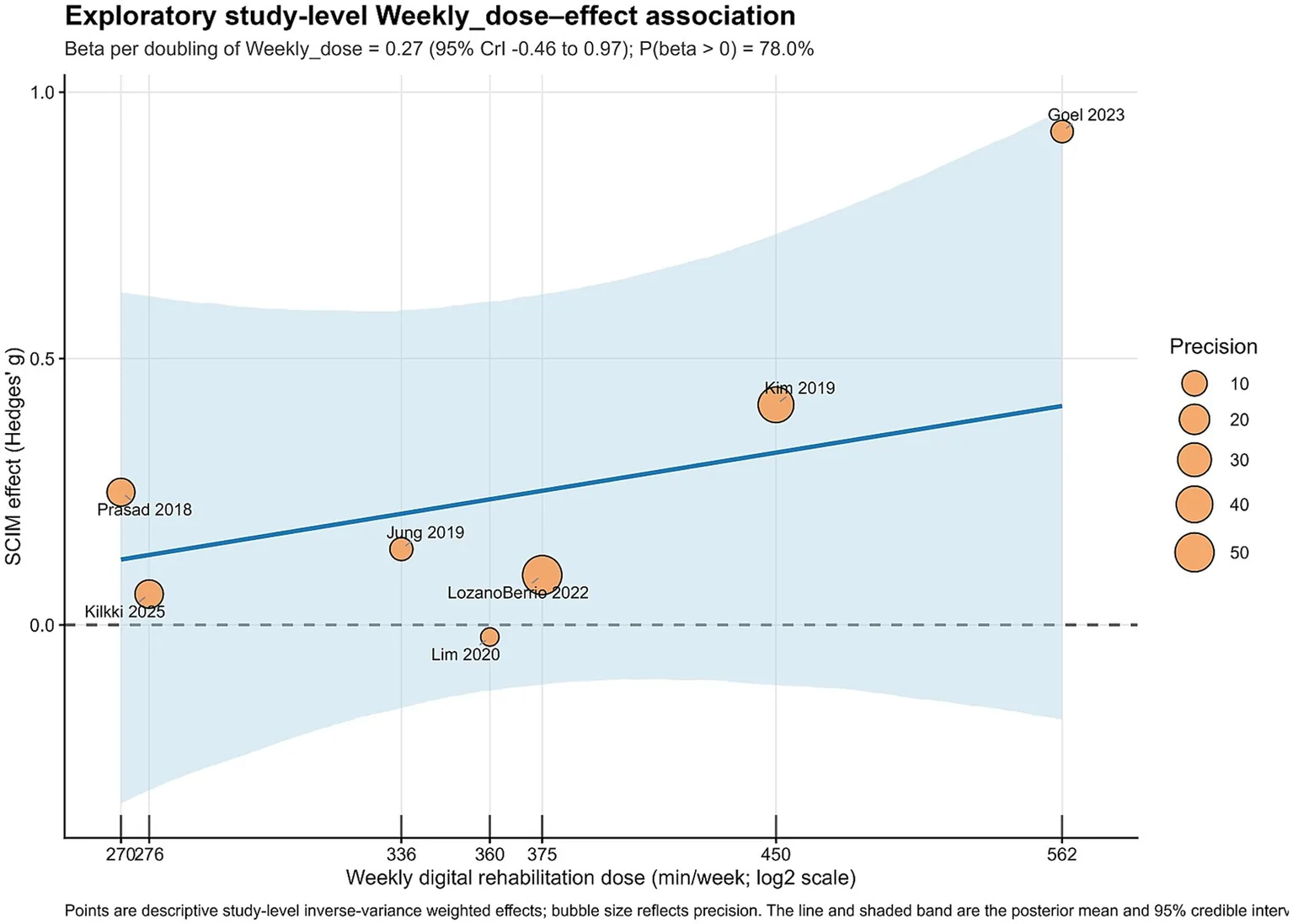
Exploratory study-level Weekly_dose–effect association. Notes: Weekly_dose is expressed as digital rehabilitation minutes per week. The regression used log2-transformed Weekly_dose; therefore, beta represents the average change in Hedges’ *g* associated with a doubling of weekly dose. Positive Hedges’ *g* favours virtual reality or gamified digital rehabilitation. This analysis is an exploratory study-level dose–effect association and should not be interpreted as a causal dose–response relationship, an optimal dose, a threshold, or a best dose.

The heterogeneity estimates changed only slightly after inclusion of the weekly-dose moderator. The posterior median of $\tau_{\text{study}}$ decreased from 0.17 (95% CrI, 0.01 to 0.68) in the model without the dose term to 0.15 (95% CrI, 0.01 to 0.66) in the dose model. The posterior median of $\tau_{\text{effect}}$ decreased from 0.55 (95% CrI, 0.10 to 0.98) to 0.53 (95% CrI, 0.08 to 0.99). The dose model did not improve out-of-sample predictive performance. Relative to the model without the dose term, the difference in expected log predictive density was $\Delta {\text{ELPD}}_{\mathrm{LOO}}=-0.35$ (SE, 1.40), and the LOOIC was 0.71 points higher for the dose model.

Across the leave-one-study-out refits, the posterior mean weekly-dose coefficient remained positive and ranged from 0.16 to 0.27. The largest absolute change from the full-data estimate was 0.11 and occurred after exclusion of the study by Goel et al. (40); however, all corresponding 95% CrIs included zero. These analyses did not provide clear evidence of a robust association between weekly dose and the SCIM effect. Moreover, because weekly dose was measured at the aggregate study level, the analysis could not establish a causal dose–response relationship or identify an optimal dose or therapeutic threshold.

### 3.8 Sensitivity and influence analyses

In the leave-one-study-out analysis of the primary SCIM model, the pooled posterior mean SMD ranged from 0.18 after exclusion of the study by Goel et al. (40) to 0.29 after exclusion of the study by Lozano-Berrio et al. (34). The maximum absolute change from the full-data estimate was 0.06 and occurred after exclusion of the study by Goel et al. (40). The posterior probability of benefit ranged from 79.9% after exclusion of the study by Kim et al. (37) to 92.7% after exclusion of the study by Goel et al. (40). All 95% CrIs continued to include zero, indicating that exclusion of no single study changed whether the interval included zero (Figure 5, Supplementary File 7).

**Figure 5:**
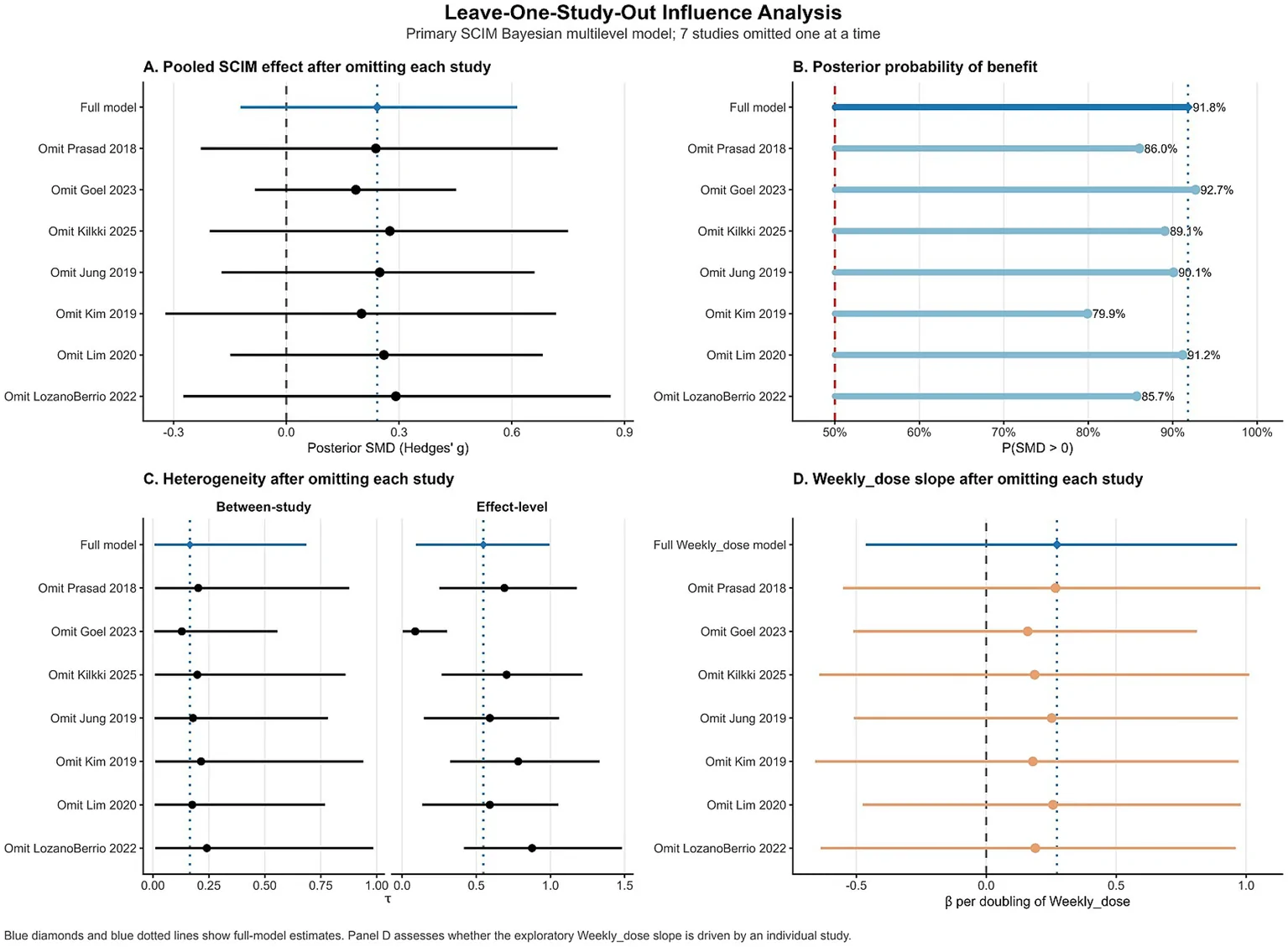
Leave-one-study-out influence analysis. Each SCIM study was omitted once and the primary Bayesian multilevel meta-analysis was refitted. The full-model estimates are shown in blue. Panel **(A)** shows the pooled SMD and 95% credible interval. Panel **(B)** shows *p*(SMD > 0). Panel **(C)** shows between-study and effect-level heterogeneity. Panel **(D)** examines whether the exploratory Weekly_dose coefficient is driven by an individual study.

In a *post hoc* sensitivity analysis based on control type, exclusion of the single SCIM study with a no-intervention control left six studies, all of which used active rehabilitation comparators. The pooled posterior mean SMD was 0.28 (95% CrI, −0.20 to 0.75), with an 89.1% posterior probability of benefit. Because the CrI remained compatible with effects favoring either group, the interpretation of the primary analysis was unchanged (Supplementary File 2.2).

The posterior median of $\tau_{\text{study}}$ ranged from 0.13 to 0.24 across the leave-one-study-out analyses. The estimate of $\tau_{\text{effect}}$ was more sensitive to the exclusion of individual studies, ranging from 0.09 after exclusion of the study by Goel et al. (40) to 0.88 after exclusion of the study by Lozano-Berrio et al. (34). The maximum absolute changes from the full-data estimates were 0.08 for $\tau_{\text{study}}$ and 0.46 for $\tau_{\text{effect}}$ . Thus, the pooled mean effect was relatively stable, whereas the estimate of effect-level heterogeneity was more sensitive to the inclusion of individual studies.

The posterior summaries were essentially unchanged under the three alternative heterogeneity-prior specifications. Under the conservative half-normal, wider study-level half-normal, and half-Student-t specifications, the pooled posterior mean SMD was 0.24 (95% CrI, −0.12 to 0.60), the posterior probability of benefit was 91.2%, and the posterior median of $\tau_{\text{study}}$ was 0.16 (95% CrI, 0.01 to 0.68) (Supplementary File 8).

The planned risk-of-bias sensitivity analysis was not estimable because all seven studies included in the SCIM analysis received an overall judgment of “some concerns,” and none was judged to be at high risk of bias. Excluding studies at high risk of bias would therefore have produced the same dataset as the full analysis.

### 3.9 Model diagnostics

The MCMC diagnostic results indicated no substantial problems with convergence or sampling efficiency. For the primary pooled SCIM effect, the rank-normalized split potential scale reduction factor was $\hat{R}=1.00$ , the bulk effective sample size was 10,343, and the tail effective sample size was 11,709. The reported $\hat{R}$ values for the seven secondary-outcome models were also 1.00, and their bulk and tail effective sample sizes were high.

Trace plots showed stable mixing across chains without apparent long-term drift, and posterior predictive density overlays generally showed adequate overlap with the distribution of the observed effect estimates. Detailed posterior summaries for the primary model parameters were as follows: pooled SMD, mean 0.242 and median 0.240 (95% CrI, −0.122 to 0.614); between-study heterogeneity, $\tau_{\text{study}}$ , mean 0.212 and median 0.165 (95% CrI, 0.007 to 0.686); and effect-level heterogeneity, $\tau_{\text{effect}}$ , mean and median both 0.548 (95% CrI, 0.093 to 0.994) (Figure 6, Supplementary File 9).

**Figure 6:**
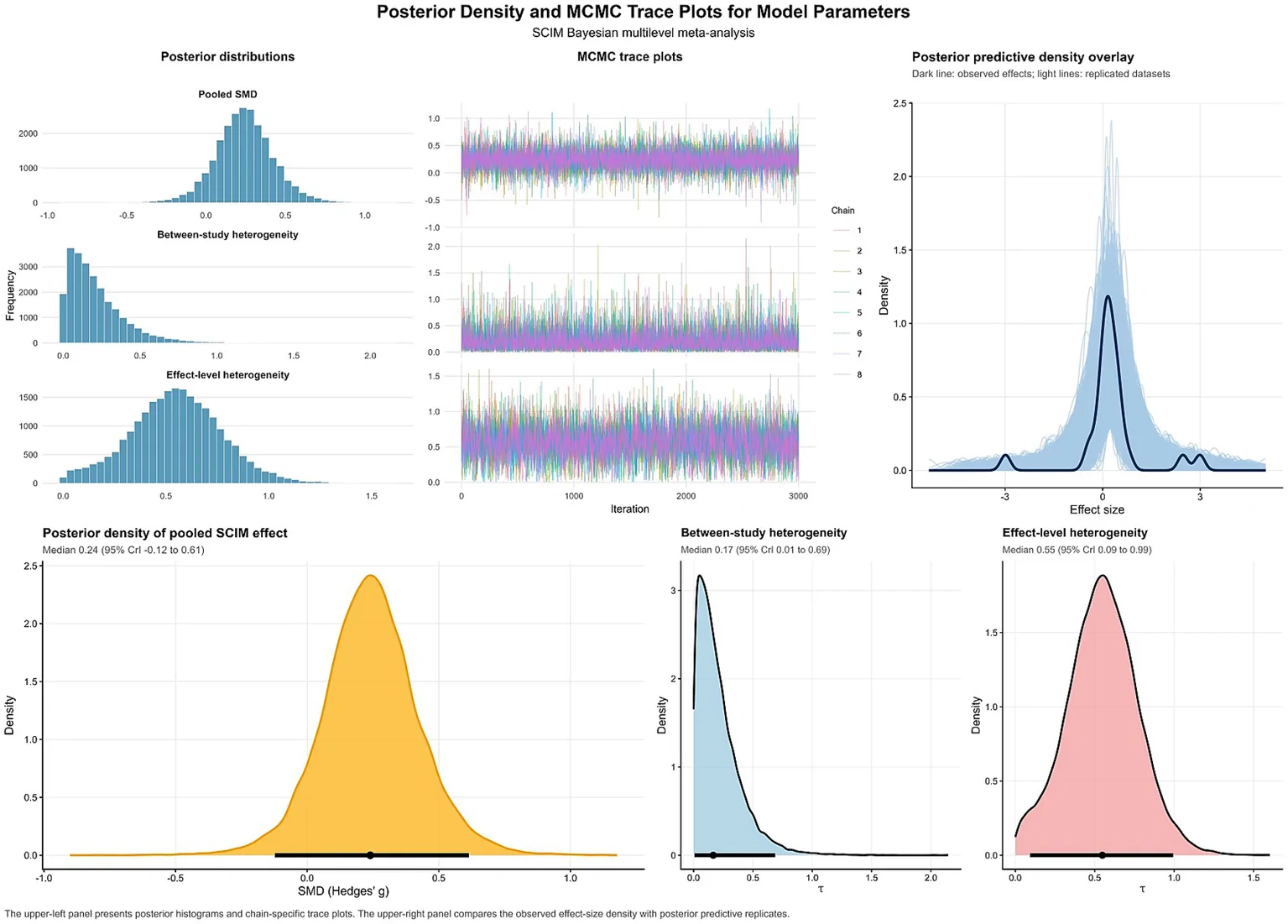
Bayesian model diagnostics for SCIM outcomes. Notes: Posterior histograms and chain-specific trace plots are shown for the principal model parameters. The posterior predictive density overlay compares the observed effect-size distribution with replicated datasets generated from the fitted model. The lower panels display the pooled SCIM posterior and the study-level and effect-level heterogeneity posteriors.

## 4 Discussion

### 4.1 Principal findings

The principal finding of this systematic review and Bayesian multilevel meta-analysis is that VR-based or gamified digital rehabilitation may confer modest average improvements in functional independence among individuals with SCI, but the current evidence does not establish that these improvements are consistently clinically important. Across 25 eligible SCIM effect estimates from seven studies, the pooled posterior mean SMD was 0.24 (95% CrI, −0.12 to 0.61). The posterior probability that the pooled effect was greater than zero was 91.8%, whereas the probability that it exceeded the prespecified distribution-based threshold for clinical importance (Hedges’ g=0.40 ) was 17.4%. The 95% PI ranged from −1.17 to 1.58, indicating substantial uncertainty in the true effect expected for a new eligible comparison in a new study and allowing for effects favoring either the intervention or the comparator. In addition, the posterior median of effect-level heterogeneity was greater than that of between-study heterogeneity, suggesting that treatment effects may vary not only across study settings and intervention-delivery approaches but also across outcomes, comparisons, and assessment conditions within individual studies.

Sensitivity and secondary analyses did not materially change this interpretation. Restricting the SCIM meta-analysis to the six studies with active-rehabilitation comparators yielded a similar but less precise posterior mean effect (SMD, 0.28; 95% CrI, −0.20 to 0.75). Posterior mean effects for all seven secondary functional outcomes favored digital rehabilitation; however, the estimates were imprecise, and all corresponding 95% CrIs included zero. Direct posterior comparisons provided no clear evidence that the SCIM effect differed by intervention category, injury completeness, or intervention duration. Likewise, the exploratory weekly-dose model did not improve out-of-sample predictive performance, and the dose–response association remained uncertain. The pooled SCIM effect was relatively stable across leave-one-study-out and alternative-prior analyses; nevertheless, the certainty of evidence was rated as very low for all outcomes. Taken together, these findings support a possible adjunctive role for digital rehabilitation in the active-care settings represented by most included trials, but they do not establish consistent, clinically important improvements or justify prioritizing a specific technology, patient subgroup, or dose regimen.

### 4.2 Comparison with previous studies

The direction of the present findings is broadly consistent with that of previous evidence, but the current analysis supports a more cautious interpretation of the magnitude and clinical importance of the benefits associated with VR-based and gamified digital rehabilitation. Previous systematic reviews have suggested that VR may improve balance, gait, or performance on specific upper-limb tasks in individuals with SCI. However, these conclusions have often been based on small studies and narrowly targeted outcomes, and some quantitative syntheses have included nonrandomized studies or single-group pre–post comparisons (10, 11, 14). By contrast, systematic reviews restricted to RCTs or focused on overall or upper-limb function have not demonstrated a consistent advantage of VR over conventional rehabilitation (12, 13, 41). The present analysis likewise did not provide clear evidence of an incremental improvement in SCIM outcomes. Nevertheless, most of the posterior mass favored a positive effect, although the estimated average effect was small and the probability of exceeding the prespecified threshold for clinical importance was low. These findings should therefore not be interpreted as evidence that digital rehabilitation is ineffective; rather, the magnitude and clinical importance of its incremental benefit for overall functional independence remain uncertain.

Differences between previous reviews and the present findings may partly reflect variation in outcome selection and study design. Measures of balance, walking speed, and specific aspects of hand function are closely aligned with the tasks practiced during training and may therefore be more responsive to digital rehabilitation. Improvements in these task-specific outcomes, however, may not translate into broader gains in independence across the domains assessed by the SCIM, including self-care, respiration and sphincter management, and mobility. In addition, some previous reviews combined randomized and nonrandomized evidence or pooled within-group pre–post changes with between-group treatment comparisons.

Comparator choice provides another source of clinical heterogeneity because each effect estimate is defined relative to a particular control condition. Of the 13 included trials, 10 used active rehabilitation comparators, two used sham or attention controls, and one used no intervention. Among the seven trials reporting SCIM outcomes, six used active rehabilitation comparators and one used no intervention. The primary pooled SCIM estimate therefore predominantly represents the incremental effect of digital rehabilitation relative to active rehabilitation, while also incorporating a single broader contrast with no intervention. Restricting the SCIM analysis to trials with active rehabilitation comparators yielded a similar but less precise estimate. Accordingly, the pooled SCIM effect should be interpreted as an average across the comparator contexts represented in the analysis, rather than as a common effect relative to any single control condition. The lack of a detectable incremental benefit in these comparisons should not be interpreted as evidence that digital rehabilitation has no therapeutic value.

A further methodological distinction of the present study was the use of a three-level Bayesian model, which retained multiple dependent effect estimates from individual studies while separately estimating between-study and effect-level heterogeneity. Posterior probabilities, a prespecified threshold for clinical importance, and a PI were used to distinguish the direction and magnitude of the average effect from uncertainty regarding future effects. This framework indicated that inconsistency in the literature may reflect not only differences between studies but also variation among outcomes, comparisons, or assessment conditions within the same study. Neither the direct posterior subgroup contrasts nor the exploratory weekly-dose meta-regression provided clear evidence that the SCIM effect varied according to intervention category, injury completeness, intervention duration, or weekly training dose. Thus, the present findings refine rather than overturn previous conclusions regarding the potential value of digital rehabilitation. Current evidence supports a possible adjunctive role but remains insufficient to establish consistent, clinically important improvements in functional independence or a hierarchy of effectiveness according to technology, patient subgroup, or training dose.

### 4.3 Clinical implications

These findings support consideration of VR-based and gamified digital rehabilitation as potential adjuncts to, rather than replacements for, standard rehabilitation. Although most of the posterior probability mass for the pooled SCIM effect favored the intervention, the probability that the effect exceeded the prespecified threshold for clinical importance was low, and the PI was wide. Of the seven trials contributing SCIM data, six used active rehabilitation comparators, and sensitivity analyses restricted to these comparators remained imprecise. The estimated effect therefore primarily represents the incremental benefit of digital rehabilitation relative to active rehabilitation and should not be interpreted as either the absolute effect versus no treatment or a common effect relative to a standardized conventional rehabilitation program. The lack of clear evidence of additional benefit does not necessarily indicate that digital rehabilitation lacks therapeutic value.

Clinical discussions should therefore communicate the possibility of modest additional functional benefits while emphasizing the substantial uncertainty and likely variation in individual responses. Current evidence does not support prioritizing VR-based rehabilitation over gamified digital rehabilitation, tailoring treatment according to injury completeness or intervention duration, or identifying an optimal weekly training dose. In the absence of a clear comparative advantage, the intervention platform and training protocol should be selected according to the targeted functional goals, feasibility of implementation, and patient preferences. Individual responses should be monitored using prespecified outcome measures and follow-up assessments. The SCIM may be used to determine whether treatment effects translate into greater independence in daily activities, whereas task-specific measures may detect changes in targeted domains of motor, hand, balance, or walking function. Given the very low certainty of evidence across all outcomes, the findings support cautious and monitored adjunctive use rather than strong recommendations, standardized prescriptions, or routine prioritization for particular patient subgroups.

### 4.4 Strengths and limitations

The principal strengths of this study include the coherence among the eligibility criteria, primary outcome selection, and statistical model. Restricting the evidence base to RCTs and designating the SCIM as the primary outcome focused the analysis on an SCI-specific measure of functional independence. The three-level Bayesian model accounted for sampling variance and the dependence among multiple effect estimates from the same study while separately estimating between-study and effect-level heterogeneity. Reporting posterior probabilities of benefit, prespecified distribution-based thresholds for clinical importance, and PIs enabled the direction and potential clinical magnitude of the average effect to be distinguished from uncertainty regarding effects in future studies. Leave-one-study-out and alternative-prior analyses further indicated that the pooled SCIM estimate was not materially influenced by any single study or by the heterogeneity-prior specifications examined.

Several limitations warrant consideration. First, the evidence base was small. The primary analysis of SCIM outcomes included only seven studies, and most secondary outcomes were reported in only three or four studies. The overall risk of bias across the 13 included studies was rated as “some concerns,” and the certainty of evidence was very low for every outcome assessed. Substantial uncertainty therefore remains regarding both the magnitude and the clinical importance of the estimated effects. Second, the included studies differed substantially in neurological injury characteristics, rehabilitation phase, digital technology, co-interventions, control condition, and training dose. Variation in control conditions is particularly important because active rehabilitation, sham or attention controls, and no-intervention controls yield different relative effect estimates and may also differ in treatment duration, therapist contact, participant expectations, and co-interventions. The SCIM evidence base consisted of six studies with active controls and one study with a no-intervention control; none used a sham or attention control. This imbalance precluded meaningful subgroup analyses by control type. Excluding the study with a no-intervention control did not materially alter the pooled effect estimate, but the confidence intervals remained wide. For secondary outcomes, only three or four studies were available, precluding robust analyses stratified by control type. Accordingly, the pooled estimates should be interpreted as average effects across the control contexts represented in the included trials, rather than as effects relative to a single common control condition. The posterior median estimate of effect-level heterogeneity was larger than the corresponding estimate of between-study heterogeneity, indicating substantial variation in effect estimates within studies across outcomes, comparisons, or assessment conditions. This heterogeneity limits the attribution of differences in effect estimates to specific intervention components, patient populations, or clinical settings and reduces the generalizability of the pooled findings.

Third, the subgroup and weekly dose analyses were based on aggregate study-level moderators from only seven studies and were therefore susceptible to ecological bias, residual confounding, and measurement error in the dose variables. Weekly dose was generally estimated from the prescribed training frequency and session duration rather than from actual training exposure, adherence, engagement, or implementation fidelity. Accordingly, the observed dose associations should not be interpreted as causal or used to establish individualized treatment rules, determine optimal doses, or define clinically meaningful dose thresholds.

## 5 Conclusion

This systematic review and Bayesian multilevel meta-analysis suggest that digital rehabilitation interventions based on VR or gamification may produce modest improvements in functional independence relative to the control conditions used in the included RCTs. However, the available evidence is insufficient to determine whether these improvements are clinically meaningful and sustained over time. Evidence for the SCIM was derived primarily from trials with active control groups, and effect estimates remained highly imprecise in analyses restricted to these trials. The available evidence also does not support prioritizing particular interventions on the basis of technology type, injury completeness, intervention duration, or training dosage. Given the very low certainty of the evidence, these interventions should currently be regarded as targeted, closely monitored adjuncts to conventional rehabilitation. Future studies should be adequately powered multicenter RCTs that use standardized, patient-important outcome measures, consistently report actual training volume, prespecify clinically meaningful control categories, and include longer-term follow-up to assess the durability of treatment effects.

## Data Availability

The original contributions presented in the study are included in the article/Supplementary material, further inquiries can be directed to the corresponding author.

## Glossary

10MWT: 10-Meter Walk Test
AIS: American Spinal Injury Association Impairment Scale
ARAT: Action Research Arm Test
ASIA-UEMS: American Spinal Injury Association Upper Extremity Motor Score
BBT: Box and Block Test
CrI: credible interval
ELPD_LOO_: expected log predictive density under leave-one-out cross-validation
GRADE: Grading of Recommendations Assessment Development and Evaluation
GRAIL: Gait Real-time Analysis Interactive Lab
K-SCIM: Korean version of the Spinal Cord Independence Measure
LOO: leave-one-out
LOOIC: leave-one-out information criterion
MCID: minimal clinically important difference
MCMC: Markov chain Monte Carlo
PI: prediction interval
PICOS: participants, interventions, comparators, outcomes, and study design
PRISMA: Preferred Reporting Items for Systematic Reviews and Meta-Analyses
PSIS-LOO: Pareto-smoothed importance-sampling leave-one-out cross-validation
RCT: randomized controlled trial
RoB 2: Cochrane Risk of Bias 2 tool
SCI: Spinal Cord Injury
SCIM: Spinal Cord Independence Measure
SCIM-III: Spinal Cord Independence Measure III
SCIM-SR: self-report Spinal Cord Independence Measure
SE: standard error
SMD: standardized mean difference
UEMS: Upper Extremity Motor Score
VR: virtual reality
WISCI: Walking Index for Spinal Cord Injury
WISCI-II: Walking Index for Spinal Cord Injury II.
