# Supplementary material for: Effects of virtual reality and gamified digital rehabilitation on functional recovery in individuals with Spinal Cord Injury: a systematic review and Bayesian multilevel meta-analysis
Source: Front Neurol. 2026 Oct 1;17:1954856. doi: 10.3389/fneur.2026.1954856 (PMC13668546; doi:10.3389/fneur.2026.1954856)
Supplement: Supplementary file 1 [file Supplementary_file_1.docx]

**Effects of Virtual Reality and Gamified Digital Rehabilitation on Functional Recovery in Individuals With Spinal Cord Injury: A Systematic Review and Bayesian Multilevel Meta-Analysis**

Supplementary Files

1: Search strategy	2

2: Characteristics of included studies	2

2.1 Comparator classification	7

2.2 Comparator-restricted sensitivity analysis for SCIM	8

3: Risk of bias	9

4: Included studies	10

5. Bayesian Subgroup Analyses of SCIM Outcomes	12

5.1 Intervention category	12

Table A. Subgroup-specific posterior estimates	12

Table B. Posterior contrast between subgroups	12

5.2 Injury completeness	12

Table A. Subgroup-specific posterior estimates	12

Table B. Posterior contrast between subgroups	12

5.3 Intervention duration	13

Table A. Subgroup-specific posterior estimates	13

Table B. Posterior contrast between subgroups	13

6. Exploratory Weekly_dose–Effect Meta-regression	14

Table 1. Weekly_dose meta-regression coefficient	14

Table 2. Heterogeneity before and after adding Weekly_dose	14

Table 3. LOO model comparison	14

Table 4. Leave-one-study-out influence analysis	14

Figure 4. Exploratory study-level Weekly_dose–effect association	15

7. Leave-One-Study-Out Influence Analysis	16

Table 1. Influence on the pooled SCIM effect	16

Table 2. Influence on posterior heterogeneity	16

Table 3. Influence on the Weekly_dose coefficient	17

Figure 5. Leave-one-study-out influence analysis	17

8 Prior and Model Sensitivity Analysis	19

Table 1. Prior sensitivity analysis	19

Table 2. Risk-of-bias sensitivity analysis	19

Figure 6. Prior and model sensitivity analysis	20

9: Posterior Density and MCMC Trace Plots for Model Parameters	21

Table 1. Posterior summaries of principal model parameters	21

Figure 7. Bayesian model diagnostics for SCIM outcomes	21

# 1: Search strategy

Embase

| 1 | 'spinal cord injury'/exp  OR ("Injuries, Spinal Cord" OR "Cord Injuries, Spinal" OR "Cord Injury, Spinal" OR "Injury, Spinal Cord" OR "Spinal Cord Injury" OR "Myelopathy, Traumatic" OR "Myelopathies, Traumatic" OR "Traumatic Myelopathies" OR "Traumatic Myelopathy" OR "Spinal Cord Trauma" OR "Cord Trauma, Spinal" OR "Cord Traumas, Spinal" OR "Spinal Cord Traumas" OR "Trauma, Spinal Cord" OR "Traumas, Spinal Cord" OR "Post-Traumatic Myelopathy" OR "Myelopathies, Post-Traumatic" OR "Myelopathy, Post-Traumatic" OR "Post-Traumatic Myelopathies" OR "Post Traumatic Myelopathy" OR "Spinal Cord Contusion" OR "Contusion, Spinal Cord" OR "Contusions, Spinal Cord" OR "Cord Contusion, Spinal" OR "Cord Contusions, Spinal" OR "Spinal Cord Contusions" OR "Spinal Cord Laceration" OR "Cord Laceration, Spinal" OR "Cord Lacerations, Spinal" OR "Laceration, Spinal Cord" OR "Lacerations, Spinal Cord" OR "Spinal Cord Lacerations" OR "Spinal Cord Transection" OR "Cord Transection, Spinal" OR "Cord Transections, Spinal" OR "Spinal Cord Transections" OR "Transection, Spinal Cord"):ti,ab,kw |
| --- | --- |
| 2 | 'robotics'/exp Or ("robot*" and "assist*"):ti,ab,kw |
| 3 | 'telemedicine'/exp OR 'smartphone'/exp OR 'mobile phone'/exp OR 'mobile application'/exp OR 'telerehabilitation'/exp OR 'clinical decision support system'/exp OR 'decision support system'/exp OR 'medical informatics'/exp OR 'web-based intervention'/exp OR 'video consultation'/exp  or ("tele*" or "Mobile*" or "app*" or "phone*" or "mHealth" or "smart?phone*" or "smart*" or "cell?phone*" or "tablet*" or "computer*" or "eHealth" or "message*" or "internet*" or "video* " or "video?conf*" or "pda" or "personal digital assistance" or "medical informatics"):ti,ab,kw |
| 4 | ("exergaming" or ((gam* OR gaming OR exer*) AND (virtual* OR virtual reality OR series* OR digital*)) or (virtual* NEAR/3 gam*) or (exer* NEAR/3 gam*) or "Xbox" or "Game*" or "exer-gaming" or "digital exercise" or "active videogame" or "Wii" or "Wii Fit" or "Kinect" or "virtual-gam*"):ti,ab,kw |
| 5 | 'virtual reality'/exp OR 'augmented reality'/exp OR 'smart-glasses'/exp OR 'augmented reality system'/exp OR 'virtual reality system'/exp OR 'clinical decision support system'/exp OR 'decision support system'/exp OR 'medical informatics'/exp OR 'web-based intervention'/exp OR 'video consultation'/exp  Or ((virtual* OR digital* OR computer* OR augment* OR immersive*)  AND (simulation OR reality OR environment) or "mixed reality synthetic environment" or "virtual-reality*" or "augmented-reality*" or "Computer-assisted" or "computer assisted"):ti,ab,kw |
| 6 | 'biofeedback'/exp OR ' Neurofeedback '/exp OR ' Sensory feedback '/exp OR 'Wearable computer'/exp OR 'Accelerometry'/exp or (feedback* or biofeedback* or wearable* or acceleromet* or gyroscop* or sensor* or shoe* or insole*):ti,ab,kw |
| 7 | 'physical stimulation'/exp or "peripheral stimulation":ti,ab,kw |

1 AND (2 or 3 or 4 or 5 or 6 or 7) AND randomised controlled trial

Cochrane

| 1 | ((Injuries, Spinal Cord" OR "Cord Injuries, Spinal" OR "Cord Injury, Spinal" OR "Injury, Spinal Cord" OR "Spinal Cord Injury" OR "Myelopathy, Traumatic" OR "Myelopathies, Traumatic" OR "Traumatic Myelopathies" OR "Traumatic Myelopathy" OR "Spinal Cord Trauma" OR "Cord Trauma, Spinal" OR "Cord Traumas, Spinal" OR "Spinal Cord Traumas" OR "Trauma, Spinal Cord" OR "Traumas, Spinal Cord" OR "Post-Traumatic Myelopathy" OR "Myelopathies, Post-Traumatic" OR "Myelopathy, Post-Traumatic" OR "Post-Traumatic Myelopathies" OR "Post Traumatic Myelopathy" OR "Spinal Cord Contusion" OR "Contusion, Spinal Cord" OR "Contusions, Spinal Cord" OR "Cord Contusion, Spinal" OR "Cord Contusions, Spinal" OR "Spinal Cord Contusions" OR "Spinal Cord Laceration" OR "Cord Laceration, Spinal" OR "Cord Lacerations, Spinal" OR "Laceration, Spinal Cord" OR "Lacerations, Spinal Cord" OR "Spinal Cord Lacerations" OR "Spinal Cord Transection" OR "Cord Transection, Spinal" OR "Cord Transections, Spinal" OR "Spinal Cord Transections" OR "Transection, Spinal Cord)):ti,ab,kw |
| --- | --- |
| 2 | ((Robot*) AND (assist*)):ti,ab,kw |
| 3 | (("telemedicine") OR ("Smartphone") OR ("Mobile phone") OR (Mobile device*) OR ("Mobile application") OR ("telerehabilitation") OR ("clinical decision support system") OR ("decision support system") OR (“medical informatics”) OR (“web-based intervention”) OR ("video-consultation") OR ("virtual consultation") OR (Tele*) OR (Mobile*) OR (application) OR (phone*) OR ("mHealth") OR (smart?phone) OR (smart*) OR (cell?phone) OR (tablet*) OR (computer*) OR ("eHealth") OR (message*) OR (internet*) OR (video*) OR (video?conf*) OR ("pda") OR ("personal digital assistance")):ti,ab,kw |
| 4 | (((("exergaming") OR (gam* OR exer* OR "gaming")) AND (virtual* OR "virtual reality" OR series* OR digital*)) OR (virtual* NEAR gam*) OR (exer* NEAR gam*) OR ("Xbox") OR (game*) OR (exer-gaming) OR ("digital exercise") OR (digit* exercis*) OR ("active videogame") OR ("wii") OR ("wii fit") OR ("kinect") OR (virtual-gam*)):ti,ab,kw |
| 5 | (("virtual reality") OR ("augmented reality") OR ("smart glasses") OR ("augmented reality system") OR ("virtual reality system") OR ("clinical decision support system") OR ("decision support system") OR ("medical informatics") OR ("web-based intervention") OR ("video consultation") OR ((virtual* OR digital* OR computer* OR augment* OR immersive*) AND ("simulation" OR "reality" OR "environment")) OR ("mixed reality synthetic environment") OR ("virtual reality") OR (augmented-reality*) OR ("computer assisted")):ti,ab,kw |
| 6 | (("biofeedback") OR ("neurofeedback") OR ("sensory-feedback") OR ("wearable computer") OR ("Accelerometry") OR (feedback*) OR (biofeedback*) OR (wearable*) OR (acceleromet*) OR (gyroscop*) OR (sensor*) OR (shoe*) OR (insole*)):ti,ab,kw |
| 7 | (("physical stimulation") OR ("peripheral stimulation")):ti,ab,kw |

1 AND (2 or 3 or 4 or 5 or 6 or 7) AND randomised controlled trial

Pubmed

| 1 | ("spinal cord injury"[Mesh])  OR "Injuries, Spinal Cord" OR "Cord Injuries, Spinal" OR "Cord Injury, Spinal" OR "Injury, Spinal Cord" OR "Spinal Cord Injury" OR "Myelopathy, Traumatic" OR "Myelopathies, Traumatic" OR "Traumatic Myelopathies" OR "Traumatic Myelopathy" OR "Spinal Cord Trauma" OR "Cord Trauma, Spinal" OR "Cord Traumas, Spinal" OR "Spinal Cord Traumas" OR "Trauma, Spinal Cord" OR "Traumas, Spinal Cord" OR "Post-Traumatic Myelopathy" OR "Myelopathies, Post-Traumatic" OR "Myelopathy, Post-Traumatic" OR "Post-Traumatic Myelopathies" OR "Post Traumatic Myelopathy" OR "Spinal Cord Contusion" OR "Contusion, Spinal Cord" OR "Contusions, Spinal Cord" OR "Cord Contusion, Spinal" OR "Cord Contusions, Spinal" OR "Spinal Cord Contusions" OR "Spinal Cord Laceration" OR "Cord Laceration, Spinal" OR "Cord Lacerations, Spinal" OR "Laceration, Spinal Cord" OR "Lacerations, Spinal Cord" OR "Spinal Cord Lacerations" OR "Spinal Cord Transection" OR "Cord Transection, Spinal" OR "Cord Transections, Spinal" OR "Spinal Cord Transections" OR "Transection, Spinal Cord" |
| --- | --- |
| 2 | ("robotics"[Mesh]) Or "robot*" and "assist*" |
| 3 | ("telemedicine"[Mesh]) OR ("smartphone"[Mesh]) OR ("mobile phone"[Mesh]) OR ("mobile application"[Mesh]) OR ("telerehabilitation"[Mesh]) OR ("clinical decision support system"[Mesh]) OR ("decision support system"[Mesh]) OR ("medical informatics"[Mesh]) OR ("web-based intervention"[Mesh]) OR ("video consultation"[Mesh])  or "tele*" or "Mobile*" or "app*" or "phone*" or "mHealth" or "smart?phone*" or "smart*" or "cell?phone*" or "tablet*" or "computer*" or "eHealth" or "message*" or "internet*" or "video* " or "video?conf*" or "pda" or "personal digital assistance" or "medical informatics" |
| 4 | ("exergaming" or ((gam* OR gaming OR exer*) AND (virtual* OR virtual reality OR series* OR digital*)) or (virtual* NEAR/3 gam*) or (exer* NEAR/3 gam*) or "Xbox" or "Game*" or "exer-gaming" or "digital exercise" or "active videogame" or "Wii" or "Wii Fit" or "Kinect" or "virtual-gam*" |
| 5 | ("virtual reality"[Mesh]) OR ("augmented reality"[Mesh]) OR ("smart-glasses"[Mesh]) OR ("augmented reality system"[Mesh]) OR ("virtual reality system"[Mesh]) OR ("clinical decision support system"[Mesh]) OR ("decision support system"[Mesh]) OR ("medical informatics"[Mesh]) OR ("web-based intervention"[Mesh]) OR ("video consultation"[Mesh])  Or (virtual* OR digital* OR computer* OR augment* OR immersive*) AND (simulation OR reality OR environment) or "mixed reality synthetic environment" or "virtual-reality*" or "augmented-reality*" or "Computer-assisted" or "computer assisted" |
| 6 | ("biofeedback"[Mesh]) OR ("Neurofeedback "[Mesh]) OR ("Sensory feedback "[Mesh]) OR ("Wearable computer"[Mesh]) OR 'Accelerometry"[Mesh])  or (feedback* or biofeedback* or wearable* or acceleromet* or gyroscop* or sensor* or shoe* or insole* |
| 7 | ("physical stimulation"[Mesh])  or "peripheral stimulation" |

1 AND (2 or 3 or 4 or 5 or 6 or 7) AND randomised controlled trial

Web of science

| 1 | (Injuries, Spinal Cord) OR (Cord Injuries, Spinal) OR (Cord Injury, Spinal) OR (Injury, Spinal Cord) OR (Spinal Cord Injury) OR (Myelopathy, Traumatic) OR (Myelopathies, Traumatic) OR (Traumatic Myelopathies) OR (Traumatic Myelopathy) OR (Spinal Cord Trauma) OR (Cord Trauma, Spinal) OR (Cord Traumas, Spinal) OR (Spinal Cord Traumas) OR (Trauma, Spinal Cord) OR (Traumas, Spinal Cord) OR (Post-Traumatic Myelopathy) OR (Myelopathies, Post-Traumatic) OR (Myelopathy, Post-Traumatic) OR (Post-Traumatic Myelopathies) OR (Post Traumatic Myelopathy) OR (Spinal Cord Contusion) OR (Contusion, Spinal Cord) OR (Contusions, Spinal Cord) OR (Cord Contusion, Spinal) OR (Cord Contusions, Spinal) OR (Spinal Cord Contusions) OR (Spinal Cord Laceration) OR (Cord Laceration, Spinal) OR (Cord Lacerations, Spinal) OR (Laceration, Spinal Cord) OR (Lacerations, Spinal Cord) OR (Spinal Cord Lacerations) OR (Spinal Cord Transection) OR (Cord Transection, Spinal) OR (Cord Transections, Spinal) OR (Spinal Cord Transections) OR (Transection, Spinal Cord) |
| --- | --- |
| 2 | (robotics) OR (robot*" and "assist*) |
| 3 | (telemedicine) OR (smartphone) OR (mobile phone) OR (mobile application) OR (telerehabilitation" OR ("clinical decision support system) OR (decision support system) OR (medical informatics) OR (web-based intervention) OR (video consultation")  or (tele*) OR (Mobile*) OR (app*) OR (phone*) OR (mHealth) OR (smart?phone*) OR (smart*) OR (cell?phone*) OR (tablet*) OR (computer*) OR (eHealth) OR (message*) OR (internet*) OR (video* ) OR (video?conf*) OR (pda) OR (personal digital assistance) OR (medical informatics) |
| 4 | (exergaming" or gam* OR gaming OR exer*) AND (virtual* OR virtual reality OR series* OR digital* or virtual* NEAR/3 gam* or exer* NEAR/3 gam* or "Xbox) OR (Game*) OR (exer-gaming) OR (digital exercise) OR (active videogame) OR (Wii) OR (Wii Fit) OR (Kinect) OR (virtual-gam*) |
| 5 | (virtual reality) OR (augmented reality) OR (smart-glasses) OR (augmented reality system) OR (virtual reality system) OR (clinical decision support system) OR (decision support system) OR (medical informatics) OR (web-based intervention) OR (video consultation) Or (virtual* OR digital* OR computer* OR augment* OR immersive*)AND (simulation OR reality OR environment or "mixed reality synthetic environment) OR (virtual-reality*) OR (augmented-reality*) OR (Computer-assisted) OR (computer assisted) |
| 6 | (biofeedback) OR (Neurofeedback ) OR (Sensory feedback ) OR (Wearable computer" OR 'Accelerometry)  or (feedback* or biofeedback* or wearable* or acceleromet* or gyroscop* or sensor* or shoe* or insole*) |
| 7 | (physical stimulation) OR (peripheral stimulation) |

1 AND (2 or 3 or 4 or 5 or 6 or 7) AND randomised controlled trial

2: Characteristics of included studies

| **No.** | **Study ID** | **Country /** **Funding** | **Study population** | **Injury characteristics** | **Intervention category /** **VR immersion** | **Intervention** | **Control / comparator** | **Device / platform** | **Rehabilitation setting** | **Training dose** | **Outcomes reported** |
| --- | --- | --- | --- | --- | --- | --- | --- | --- | --- | --- | --- |
| **1** | **Kowalczewski_2011** | **Country:** Canada  **Funding:** Y | Adults with chronic C5-C7 tetraplegia (≥9 months post-SCI) and residual motor function sufficient to place the hand on a table and augment grasp/release with FES; 13 unique participants contributed 18 treated hands (10 right, 8 left), including 5 participants who later repeated the crossover protocol with the other hand. | **Level:** C5-C7  **Completeness:** mixed (4/13 complete; 9/13 incomplete)  **AIS grade:** not reported  **Phase:** chronic | **Category:** Game-based digital rehabilitation  **VR immersion:** Non-immersive | ReJoyce ET: FES-assisted, task-oriented in-home tele-rehabilitation on the Rehabilitation Joystick for Computerized Exercise (ReJoyce) workstation; custom computer games simulated ADL-related grasp, release, reaching, pronation/supination, key grip, pouring, weeding, target shooting, driving, catching, and boxing. | Conventional in-home tele-rehabilitation: 20 min strength training with weighted wristlets and/or Powerweb, 20 min accuracy training using a trackball to play computer games, and 20 min cyclical therapeutic electrical stimulation (TES). | ReJoyce spring-loaded instrumented workstation with ADL manipulanda + 3-channel FES garment triggered by tooth-click sensor; laptop, webcam, VNC, and Skype for tele-supervision. | Home-based in-home tele-rehabilitation; remote therapist supervision; laboratory outcome testing. | **Duration:** 6 weeks  **Frequency:** 5 sessions/week  **Session:** 60 min | **Grip strength; Pinch strength; ARAT** |
| **2** | **Prasad_2018** | **Country:** India  **Funding:** N | Adults 18–55 years with traumatic cervical SCI/tetraplegia, complete or incomplete motor paralysis at C5–C8, at least 6 months post-injury, medically stable and able to hold a 2.54-cm block with the target hand | **Level:** C5–C7  **Completeness:** mixed  **AIS grade:** mixed (AIS A–D)  **Phase:** mixed/author-described subacute and chronic; all participants ≥6 months post-SCI | **Category:** VR/virtual environment  **VR immersion:** Non-immersive | Nintendo Wii non-immersive VR + conventional occupational therapy | Conventional hand therapy alone: target-upper-limb strength training (active or active-assisted movements) and functional tasks including reaching, moving checkers, grasping/releasing, manipulation and rotation | Nintendo Wii with Wii Sports Resort and Wiimote; games: Table Tennis, Swordplay Speed Slice, Bowling, and Cycling | Institutional hospital rehabilitation (inpatient and outpatient), Indian Spinal Injuries Centre, New Delhi, India | **Duration:** 4 weeks  **Frequency:** 3 sessions/week  **Session:** 30 min | **SCIM; BBT** |
| **3** | **Jung_2019** | **Country:** Korea  **Funding:** Y | Hospitalized adults with tetraplegic cervical SCI; motor level C2–C8; AIS A–D; <24 months post-injury | **Level:** C2-C7  **Completeness:** mixed  **AIS grade:** mixed (AIS A–D)  **Phase:** subacute | **Category:** Game-based digital rehabilitation  **VR immersion:** Non-immersive | combined upper limb robotic therapy (ArmeoPower + Amadeo) + conventional OT/daily inpatient rehabilitation | Conventional occupational therapy (OT) + daily inpatient rehabilitation program | ArmeoPower (Hocoma Inc.) + Amadeo (Tyromotion Inc.); game-based robotic upper-limb training | Inpatient rehabilitation, National Rehabilitation Center, Seoul, Korea | **Duration:** 5 weeks  **Frequency:** 3 sessions/week  **Session:** 40 min | **SCIM; UEMS; Grip strength; Pinch strength** |
| **4** | **Kim_2019** | **Country:** Korea  **Funding:** Y | Adults >19 years with tetraplegia due to SCI; <1 year since injury; at least trace strength in >=1 upper-extremity key muscle; inpatient rehabilitation | **Level:** C2-C8  **Completeness:** mixed  **AIS grade:** mixed (AIS A/B/C/D = 4/3/2/8 per arm)  **Phase:** mixed (acute/subacute; all <1 year post-injury) | **Category:** Game-based digital rehabilitation  **VR immersion:** Non-immersive | Armeo Power upper-limb robotic therapy + shared usual inpatient rehabilitation | Additional conventional occupational therapy, 30 min/session, 5 sessions/week for 4 weeks; both groups also received the same usual inpatient PT/OT | Armeo Power (Hocoma, Switzerland); exoskeleton upper-extremity robot with grip module and audio-visual Functional Exercise Programs | Institutional inpatient rehabilitation hospital, National Rehabilitation Center, Seoul, Korea | **Duration:** 4 weeks  **Frequency:** 5 sessions/week  **Session:** 30 min | **SCIM; UEMS** |
| **5** | **Lim_2020** | **Country:** Korea  **Funding:** Y | Adults 19–75 years with upper limb dysfunction and incomplete motor paralysis due to SCI at C4–C8; <12 months since onset; sufficient hand grip to hold controller; able to sit alone | **Level:** C4–C6  **Completeness:** incomplete  **AIS grade:** AIS-D  **Phase:** acute/subacute (<12 months since onset; mean around 1 month) | **Category:** VR/virtual environment  **VR immersion:** Fully immersive | fully immersive VR training + conventional rehabilitation | Conventional rehabilitation alone: 30 min occupational therapy plus 30 min ADL training, 4 days/week for 4 weeks | HTC VIVE VR RehabWare (Tech Village Corp., Goyang, Korea); precision controllers, HMD headset, trackers/base stations; six VR tasks | Hospital/inpatient rehabilitation; Chungnam National University Hospital, Daejeon, Korea | **Duration:** 4 weeks  **Frequency:** 4 sessions/week  **Session:** 30 min | **SCIM; BBT; UEMS; Grip strength; Pinch strength; ARAT** |
| **6** | **An_2022** | **Country:** Korea  **Funding:** N | Adults with chronic motor-incomplete cervical SCI (C5–C7; AIS C–D), quadriceps MMT >3, lower-extremity weight-bearing in daily life, and able to perform the 10MWT; home-based rehabilitation trial. | **Level:** C5-C7  **Completeness:** incomplete  **AIS grade:** mixed (AIS C/D = 7/13)  **Phase:** chronic (>1 year post-injury) | **Category:** VR/virtual environment  **VR immersion:** Fully immersive | HMD-based virtual soccer game rehabilitation with seated bilateral knee-extension training | Same-dose seated bilateral knee-extension rehabilitation without VR content, guided by a certified physical therapist | Head-mounted display (HMD), lower-limb motion-recognition input devices (Arduino + bilateral gyroscopes), base station, and avatar-based virtual soccer/iron-ball game | Home-based rehabilitation in participants' homes, Gyeonggi, Korea | **Duration:** 4 weeks  **Frequency:** 3 sessions/week  **Session:** 30 min | **10MWT** |
| **7** | **LozanoBerrio_2022** | **Country:** Spain  **Funding:** N | Patients aged 16-75 years with subacute cervical SCI (<6 months), traumatic or non-progressive medical etiology, AIS A-D, and able to maintain a seated posture; inpatient rehabilitation. | **Level:** C2-C7  **Completeness:** mixed  **AIS grade:** mixed (AIS A/B/C/D = 3/3/1/6)  **Phase:** subacute (<6 months post-injury) | **Category:** Game-based digital rehabilitation  **VR immersion:** Non-immersive | Armeo Spring upper-limb robotic therapy: 15 min standardized Armeo games + 15 min virtual ADL drinking, plus shared 30 min conventional therapy | Active comparator: both groups received 30 min conventional upper-limb functional/ADL therapy; the control arm received an additional 30 min conventional therapy, whereas the intervention arm received 30 min Armeo Spring robotic therapy; 5 sessions/week for 8 weeks, 40 sessions. | Armeo Spring (Hocoma AG, Switzerland); spring-based weight-compensation upper-limb exoskeleton with pressure-sensitive handgrip; Armeo software games and a custom virtual drinking application developed with VICOMTECH | Institutional inpatient rehabilitation, National Hospital for Spinal Cord Injury, Toledo, Spain | **Duration:** 8 weeks  **Frequency:** 5 sessions/week  **Session:** 30 min | **SCIM** |
| **8** | **Sanders_2022** | **Country:** USA  **Funding:** Y | Adults aged 18–80 years with chronic SCI affecting hand function for at least 6 months, able to transfer at least 3 blocks on the BBT with the training hand, with no other active major neurological disease and upper-limb pain ≤3/10; 10 participants completed the randomized crossover study. | **Level:** not reported  **Completeness:** incomplete  **AIS grade:** AIS C/D (C=1, D=4)  **Phase:** chronic (≥6 months post-SCI) | **Category:** Game-based digital rehabilitation  **VR immersion:** Non-immersive | MusicGlove — EOT1, end of the first 3-week intervention (MusicGlove-first sequence). Exact change score reported in the article; endpoint values converted with the supplied baseline/change-to-endpoint formula using R=1. | Conventional home hand-exercise booklet: 18 standard exercises developed by an occupational therapist; 2 sets twice daily with activity-specific repetitions and hold times; prescribed minimum 3 h/week over at least 3 sessions/week for 3 weeks. | MusicGlove wearable grip-sensor glove connected to a computer-based music game; six electrical leads detect five functional grip types and provide visual/auditory game feedback; no robotic assistance. | Home-based self-guided hand rehabilitation; participants' homes | **Duration:** 3 weeks  **Frequency:** 3 sessions/week  **Session:** 60 min | **BBT** |
| **9** | **Goel_2023** | **Country:** India  **Funding:** N | Adults 18–60 years with acute/sub-acute incomplete SCI/paraplegia (AIS B–D), able to sit unsupported ≥10 s; duration of injury <10 months | **Level:** T6–L2  **Completeness:** incomplete  **AIS grade:** mixed (AIS B–D)  **Phase:** acute/sub-acute | **Category:** VR/virtual environment  **VR immersion:** Fully immersive | immersive VR + CPT | Functional electrical stimulation (FES) to rectus abdominis and erector spinae + conventional physical therapy (CPT) | Ocular Grand VR spectacles (Ocular, Aztek Hub LLP, Mohali, Punjab, India) with mobile phone; games: Roller Coaster VR, In Mind VR, VR Tunnel Race | Neurological Physiotherapy Out Patient Department, tertiary care hospital | **Duration:** 4 weeks  **Frequency:** 5 sessions/week  **Session:** 45 min | **SCIM** |
| **10** | **MollaCasanova_2024** | **Country:** Spain  **Funding:** Y | Adults with chronic motor-incomplete SCI (AIS C–D), >1 year post-injury, able to walk with or without aids and understand instructions; recruited through a hospital service and disability associations. | **Level:** C4-C7; C5-D1; L3; L5-S1  **Completeness:** incomplete  **AIS grade:** mixed (AIS C/D = 2/4)  **Phase:** chronic (>1 year post-injury) | **Category:** VR/virtual environment  **VR immersion:** Non-immersive | Virtual Walking visual-illusion therapy (10 min) + shared therapeutic exercise (35 min: gait coordination, strength, balance, and stretching) | Placebo Virtual Walking using the same mirror/standing-frame/screen setup with a landscape video without human or animal movement + the same 35-min therapeutic exercise program. | Non-immersive Virtual Walking setup: mirror for the upper body, standing frame/ad hoc lower-body support, and screen projecting treadmill-walking legs adapted to participant height and weight | Hospital service and disability associations (Hospital Universitario y Politécnico La Fe; TetraSport, CODIFIVA and ASPAYM), Spain | **Duration:** 6 weeks  **Frequency:** 3 sessions/week  **Session:** 10 min | **10MWT; WISCI** |
| **11** | **Kilkki_2025** | **Country:** Finland  **Funding:** Y | Adults 34-73 y, 1-8 years after incomplete cervical SCI; all randomized participants AIS D; technology-assisted upper-extremity rehabilitation study. | **Level:** C2-C8  **Completeness:** incomplete  **AIS grade:** AIS D  **Phase:** chronic | **Category:** Game-based digital rehabilitation  **VR immersion:** Non-immersive | Technology-assisted upper extremity rehabilitation (AMADEO/DIEGO/PABLO) | 6 weeks of no intervention; no other physical rehabilitation during the trial. | AMADEO, DIEGO, PABLO (Tyromotion GmbH, Graz, Austria) | Institutional setting (Validia Rehabilitation Center / private outpatient neurorehabilitation clinic, Helsinki) | **Duration:** 6 weeks  **Frequency:** 3 sessions/week  **Session:** 40 min | **SCIM; UEMS; Grip strength; Pinch strength; ARAT** |
| **12** | **Zhao_2025** | **Country:** China  **Funding:** NR | Adults with chronic thoracic AIS A SCI (T9-T12; >12 months since injury). Mid-assessment available-case WANR cohort n=10, including P1 who withdrew after the 5-month assessment. Age was reported only in 5-year bands; mean_age/sd_age are estimates from band midpoints. Paper flow is internally inconsistent (Methods: 24 randomized with 3 dropouts/arm; Table 1: WANR n=10 and NR n=9). | **Level:** T9-T12  **Completeness:** complete  **AIS grade:** AIS A  **Phase:** chronic (>12 months post-SCI) | **Category:** VR/virtual environment  **VR immersion:** Fully immersive | Modified Walk Again Neurorehabilitation Protocol (WANR): 2 sessions/week of 45-min EEG-based non-invasive BMI with immersive VR motor imagery followed by 40-min Lokomat robotic gait training, alternating with 2 sessions/week of approximately 60-min SkyTrack body-weight-supported overground walking; active-time average = 72.5 min per scheduled session (10-20 min rest excluded). | Classical neurorehabilitation: passive stretching, assisted standing, transfers, and other individualized rehabilitation exercises, performed 4 times/week for 36 weeks; home-based after instruction, with remote oversight by telephone/WeChat. | 16-channel EEG system (Brain Products) + Oculus Rift immersive HMD; Lokomat robotic treadmill (Hocoma AG); SkyTrack 800 robotic body-weight-support overground system (Humaneotec), KAFO and walker. | Single-center institutional neurorehabilitation laboratory, Xuanwu Hospital, Beijing, China | **Duration:** 36 weeks  **Frequency:** 4 sessions/week  **Session:** 72.5 min | **WISCI** |
| **13** | **MollaCasanova_2026** | **Country:** Spain  **Funding:** Y | Adults >18 years with chronic motor-incomplete SCI (AIS C–D), cervical, thoracic, or lumbar injury, ≥6 months post-injury, able to walk at least 10 m with or without walking aids, and able to understand instructions (MMSE >23); recruited from hospitals, disability associations, and a clinic in Spain and France. | **Level:** mixed (cervical/thoracic/lumbar = 6/8/5)  **Completeness:** incomplete  **AIS grade:** mixed (AIS C/D = 8/11)  **Phase:** chronic (≥6 months post-injury) | **Category:** VR/virtual environment  **VR immersion:** Non-immersive | Virtual Walking visual-illusion therapy (10 min) + shared therapeutic physical exercise (30 min: gait coordination, balance, strength, and stretching) | Placebo Virtual Walking using the same standing-support/mirror/screen setup with videos without human or animal movement + the same 30-min therapeutic physical exercise program. | Non-immersive Virtual Walking setup: specially designed standing-support system, mirror reflecting the upper body, and height-adjustable screen projecting body-size-matched moving legs | Exercise laboratory; participants recruited through hospitals, SCI/disability associations, and a clinic in Spain and France | **Duration:** 6 weeks  **Frequency:** 3 sessions/week  **Session:** 10 min | **10MWT; WISCI** |

**Sample Sizes in the Intervention and Control Groups Across the 13 Included Studies (Total N = 295)**

| **No.** | **Study** | **Study Design** | **Intervention (Experimental) Group / Sequence A, n** | **Control Group / Sequence B, n** | **Total, n** |
| --- | --- | --- | --- | --- | --- |
| 1 | Prasad et al., 2018 | RCT | 11 | 9 | 20 |
| 2 | Sanders et al., 2022 | RCT | 5 | 5 | 10 |
| 3 | An et al., 2022 | RCT | 20 | 20 | 40 |
| 4 | Goel et al., 2023 | RCT | 9 | 9 | 18 |
| 5 | Jung et al., 2019 | RCT | 17 | 13 | 30 |
| 6 | Kilkki et al., 2025 | RCT | 9 | 7 | 16 |
| 7 | Kim et al., 2019 | RCT | 17 | 17 | 34 |
| 8 | Kowalczewski et al., 2011 | RCT | 6 | 7 | 13 |
| 9 | Lim et al., 2020 | RCT | 10 | 10 | 20 |
| 10 | Lozano-Berrio et al., 2022 | RCT | 13 | 13 | 26 |
| 11 | Mollà-Casanova et al., 2024 | RCT | 6 | 6 | 12 |
| 12 | Mollà-Casanova et al., 2026 | RCT | 19 | 19 | 38 |
| 13 | Zhao et al., 2025 | RCT | 9 | 9 | 18 |
| **Total** | | | **151** | **144** | **295** |

**Number of Included Studies and Participants for Each of the Eight Outcome Measures**

| **No.** | **Outcome Measure** | **Included Studies and Sample Sizes, n** | **Number of Studies, k** | **Total Participants, n** |
| --- | --- | --- | --- | --- |
| 1 | SCIM total score (SCIM, SCIM-III, K-SCIM, or SCIM-SR) | Prasad (20); Goel (18); Jung (30); Kilkki (16); Kim (34); Lim (20); Lozano-Berrio (26) | 7 | **164** |
| 2 | Box and Block Test (BBT) | Prasad (20); Sanders (10); Lim (20) | 3 | **50** |
| 3 | Upper Extremity Motor Score (UEMS / ASIA-UEMS) | Jung (30); Kilkki (16); Kim (34); Lim (20) | 4 | **100** |
| 4 | Grip strength | Jung (30); Kilkki (16); Kowalczewski (13); Lim (20) | 4 | **79** |
| 5 | Pinch strength | Jung (30); Kilkki (16); Kowalczewski (13); Lim (20) | 4 | **79** |
| 6 | 10-Meter Walk Test (10MWT) | An (40); Mollà-Casanova 2024 (12); Mollà-Casanova 2026 (38) | 3 | **90** |
| 7 | Walking Index for Spinal Cord Injury (WISCI / WISCI-II) | Mollà-Casanova 2024 (12); Mollà-Casanova 2026 (38); Zhao (18) | 3 | **68** |
| 8 | Action Research Arm Test (ARAT) | Kilkki (16); Kowalczewski (13); Lim (20) | 3 | **49** |

# 2.1 Comparator classification

Comparator conditions were classified according to the clinical contrast represented by the control group. Active rehabilitation comparators delivered conventional therapy, a dose-matched non-digital task, or another active rehabilitation intervention; sham or attention controls reproduced the intervention setting or attention while withholding the intended therapeutic digital content; and minimal controls comprised wait-list or no intervention. The classification describes the comparator type and does not imply equivalence in treatment dose, therapist contact, expectations, background rehabilitation, or co-interventions.

**Table S2.1. Comparator classification and trial-level contrast.**

| **Study** | **Comparator condition** | **Category** | **Dose, contact, and background-care features** | **Outcomes contributed** |
| --- | --- | --- | --- | --- |
| Kowalczewski et al., 2011 | Conventional in-home telerehabilitation: strengthening, accuracy training, and cyclical therapeutic electrical stimulation | Active rehabilitation | Session duration and schedule broadly matched; intervention content differed | Grip strength; pinch strength; ARAT |
| Prasad et al., 2018 | Conventional hand therapy alone | Active rehabilitation | Conventional therapy provided in both arms; exact matching of total treatment time was not clearly reported | SCIM; BBT |
| Jung et al., 2019 | Conventional occupational therapy plus daily inpatient rehabilitation | Active rehabilitation | Usual rehabilitation in both arms; gamified robotic therapy added in the intervention arm | SCIM; UEMS; grip strength; pinch strength |
| Kim et al., 2019 | Additional conventional occupational therapy plus shared usual inpatient rehabilitation | Active rehabilitation | Additional 30-min sessions and background rehabilitation were matched | SCIM; UEMS |
| Lim et al., 2020 | Conventional rehabilitation alone: occupational therapy and activities-of-daily-living training | Active rehabilitation | Conventional rehabilitation in both arms; VR training added in the intervention arm | SCIM; BBT; UEMS; grip strength; pinch strength; ARAT |
| An et al., 2022 | Therapist-guided seated bilateral knee-extension training without VR content | Active rehabilitation | Training task, session duration, and schedule matched | 10MWT |
| Lozano-Berrio et al., 2022 | Additional conventional upper-limb functional/activities-of-daily-living therapy plus shared conventional therapy | Active rehabilitation | Additional therapy time and shared background therapy matched | SCIM |
| Sanders et al., 2022 | Conventional home hand-exercise booklet | Active rehabilitation | Home practice dose was prescribed in both arms; intervention content and feedback differed | BBT |
| Goel et al., 2023 | Functional electrical stimulation of trunk muscles plus conventional physical therapy | Active rehabilitation | Both arms received conventional therapy; active treatment content differed | SCIM |
| Mollà-Casanova et al., 2024 | Placebo virtual walking using the same apparatus with a landscape video, plus the same therapeutic exercise | Sham/attention control | Setting, attention, and background exercise matched | 10MWT; WISCI |
| Kilkki et al., 2025 | No intervention and no other physical rehabilitation during the trial | No intervention | No treatment-time or therapist-contact matching | SCIM; UEMS; grip strength; pinch strength; ARAT |
| Zhao et al., 2025 | Classical neurorehabilitation: stretching, assisted standing, transfers, and individualized exercises | Active rehabilitation | Scheduled frequency broadly matched; content and delivery differed | WISCI |
| Mollà-Casanova et al., 2026 | Placebo virtual walking using the same apparatus with videos without human or animal movement, plus the same therapeutic exercise | Sham/attention control | Setting, attention, and background exercise matched | 10MWT; WISCI |

# 2.2 Comparator-restricted sensitivity analysis for SCIM

The primary SCIM synthesis included six studies with active-rehabilitation comparators and one study with a no-intervention comparator; no SCIM study used a sham or attention control. A formal comparator-category moderator analysis was not estimable as a meaningful between-category comparison because the non-active comparator category contained only one study. We therefore restricted a post hoc sensitivity analysis to the six active-comparator studies.

**Table S2.2. Comparator-restricted sensitivity analysis for SCIM outcomes.**

| **Analysis** | **Studies** | **Pooled SMD** | **95% CrI** | **Pr(SMD > 0)** | **Between-study τ, median (95% CrI)** | **Effect-level τ, median (95% CrI)** |
| --- | --- | --- | --- | --- | --- | --- |
| Primary model: all eligible comparators | 7 | 0.24 | [−0.12, 0.61] | 91.8% | 0.17 [0.01, 0.69] | 0.55 [0.09, 0.99] |
| Post hoc sensitivity: active-rehabilitation comparators only | 6 | 0.28 | [−0.20, 0.75] | 89.1% | 0.20 [0.01, 0.86] | 0.70 [0.27, 1.22] |

Notes: Positive SMD values favor the intervention; both 95% CrIs included zero.

# 3: Risk of bias

**Figure 1. Risk of bias assessment of the included studies using the Cochrane Risk of Bias 2 tool.**
(A) Traffic-light plot showing the domain-specific and overall risk-of-bias judgments for each included study. (B) Percentage distribution of risk-of-bias judgments across the five domains and the overall assessment.

# 4: Included studies

[1–13]

1. Zhao G-G, Alho EJL, Wei P-H, Tang Y, Liu L, Zhu L, et al. Training with a non-invasive brain-machine interface, combined with virtual reality and assisted robotic locomotion, induces significant motor recovery and partial reversal of widespread cortical atrophy in asia a paraplegics: A randomized, controlled, single-center trial. https://doi.org/10.64898/2025.11.28.25340891

2. Sanders Q, Chan V, Augsburger R, Cramer SC, Reinkensmeyer DJ, Sharp K. Feasibility of home hand rehabilitation using musicglove after chronic spinal cord injury. Spinal Cord Ser Cases. 2022;8:86. https://doi.org/10.1038/s41394-022-00552-4

3. Prasad S, Aikat R, Labani S, Khanna N. Efficacy of virtual reality in upper limb rehabilitation in patients with spinal cord injury: A pilot randomized controlled trial. Asian Spine J. 2018;12:927–34. https://doi.org/10.31616/asj.2018.12.5.927

4. Mollà-Casanova S, Muñoz-Gómez E, Sempere-Rubio N, Moreno-Segura N, Inglés M, González L-M, et al. Effect of a virtual walking and therapeutic exercise-based intervention on gait and balance in people with incomplete spinal cord injury. PM&R. 2026;pmrj.70140. https://doi.org/10.1002/pmrj.70140

5. Mollà-Casanova S, Muñoz-Gómez E, Aguilar-Rodríguez M, Inglés M, Sempere-Rubio N, Moreno-Segura N, et al. Effectiveness of virtual-walking intervention combined with exercise on improving pain and function in incomplete spinal cord injury: A feasibility study. Spinal Cord Ser Cases. 2024;10:64. https://doi.org/10.1038/s41394-024-00675-w

6. Lozano-Berrio V, Alcobendas-Maestro M, Polonio-López B, Gil-Agudo A, De La Peña-González A, De Los Reyes-Guzmán A. The impact of robotic therapy on the self-perception of upper limb function in cervical spinal cord injury: A pilot randomized controlled trial. Int J Environ Res Public Health. 2022;19:6321. https://doi.org/10.3390/ijerph19106321

7. Lim DY, Hwang DM, Cho KH, Moon CW, Ahn SY. A fully immersive virtual reality method for upper limb rehabilitation in spinal cord injury. Ann Rehabil Med. 2020;44:311–9. https://doi.org/10.5535/arm.19181

8. Kowalczewski J, Chong SL, Galea M, Prochazka A. In-home tele-rehabilitation improves tetraplegic hand function. Neurorehabil Neural Repair. 2011;25:412–22. https://doi.org/10.1177/1545968310394869

9. Kim J, Lee BS, Lee H-J, Kim H-R, Cho D-Y, Lim J-E, et al. Clinical efficacy of upper limb robotic therapy in people with tetraplegia: A pilot randomized controlled trial. Spinal Cord. 2019;57:49–57. https://doi.org/10.1038/s41393-018-0190-z

10. Kilkki MM, Poutanen J, Kauranen K, Arokoski J, Hiekkala S. Effects of technology-assisted rehabilitation after spinal cord injury: Pilot randomized controlled crossover trial. JMIR Rehabil Assistive Technol. 2025;12:e78091–e78091. https://doi.org/10.2196/78091

11. Jung JH, Lee HJ, Cho DY, Lim J-E, Lee BS, Kwon SH, et al. Effects of combined upper limb robotic therapy in patients with tetraplegic spinal cord injury. Ann Rehabil Med. 2019;43:445–57. https://doi.org/10.5535/arm.2019.43.4.445

12. Goel T, Sharma N, Gehlot A, Srivastav AK. Effectiveness of immersive virtual reality training to improve sitting balance control among individuals with acute and sub-acute paraplegia: A randomized clinical trial. J Spinal Cord Med. 2023;46:964–74. https://doi.org/10.1080/10790268.2021.2012053

13. An Y, Park C. The effects of virtual soccer game on balance, gait function, and kick speed in chronic incomplete spinal cord injury: A randomized controlled trial. Spinal Cord. 2022;60:504–9. https://doi.org/10.1038/s41393-021-00745-y

# 5. Bayesian Subgroup Analyses of SCIM Outcomes

All estimates are expressed as Hedges' g. Positive values favour virtual reality or gamified digital rehabilitation. Evidence of effect modification is based on posterior subgroup contrasts.

# 5.1 Intervention category

## Table A. Subgroup-specific posterior estimates

| **Group** | **Studies** | **Effects** | **Posterior SMD** | **95% CrI** | **Pr(SMD > 0)** |
| --- | --- | --- | --- | --- | --- |
| Game-based digital rehabilitation | 4 | 18 | 0.18 | [-0.30, 0.64] | 80.8% |
| VR virtual environment | 3 | 7 | 0.36 | [-0.31, 1.02] | 87.4% |

## Table B. Posterior contrast between subgroups

| **Contrast** | **Difference in SMD** | **95% CrI** | **Pr(difference > 0)** | **Pr(difference < 0)** |
| --- | --- | --- | --- | --- |
| VR virtual environment minus Game-based digital rehabilitation | 0.19 | [-0.63, 0.98] | 69.1% | 30.9% |

Notes: Positive subgroup-specific SMD values favour virtual reality or gamified digital rehabilitation. Evidence of effect modification should be based on the posterior contrast. A positive contrast means the second subgroup listed has a larger estimated effect than the first subgroup.

# 5.2 Injury completeness

## Table A. Subgroup-specific posterior estimates

| **Group** | **Studies** | **Effects** | **Posterior SMD** | **95% CrI** | **Pr(SMD > 0)** |
| --- | --- | --- | --- | --- | --- |
| Mixed | 4 | 17 | 0.21 | [-0.29, 0.69] | 83.8% |
| Incomplete | 3 | 8 | 0.28 | [-0.38, 0.92] | 81.7% |

## Table B. Posterior contrast between subgroups

| **Contrast** | **Difference in SMD** | **95% CrI** | **Pr(difference > 0)** | **Pr(difference < 0)** |
| --- | --- | --- | --- | --- |
| Incomplete minus Mixed | 0.06 | [-0.75, 0.88] | 56.6% | 43.4% |

Notes: Positive subgroup-specific SMD values favour virtual reality or gamified digital rehabilitation. Evidence of effect modification should be based on the posterior contrast. A positive contrast means the second subgroup listed has a larger estimated effect than the first subgroup.

# 5.3 Intervention duration

## Table A. Subgroup-specific posterior estimates

| **Group** | **Studies** | **Effects** | **Posterior SMD** | **95% CrI** | **Pr(SMD > 0)** |
| --- | --- | --- | --- | --- | --- |
| ≤4 weeks | 4 | 12 | 0.39 | [-0.14, 0.88] | 93.8% |
| >4 weeks | 3 | 13 | 0.09 | [-0.44, 0.61] | 65.2% |

## Table B. Posterior contrast between subgroups

| **Contrast** | **Difference in SMD** | **95% CrI** | **Pr(difference > 0)** | **Pr(difference < 0)** |
| --- | --- | --- | --- | --- |
| >4 weeks minus ≤4 weeks | -0.30 | [-1.03, 0.44] | 17.9% | 82.0% |

Notes: Positive subgroup-specific SMD values favour virtual reality or gamified digital rehabilitation. Evidence of effect modification should be based on the posterior contrast. A positive contrast means the second subgroup listed has a larger estimated effect than the first subgroup.

# 6. Exploratory Weekly_dose–Effect Meta-regression

## Table 1. Weekly_dose meta-regression coefficient

| **Parameter** | **Beta** | **95% CrI** | **Pr(beta > 0)** | **Pr(beta < 0)** | **Studies** | **Effects** |
| --- | --- | --- | --- | --- | --- | --- |
| Weekly_dose per doubling | 0.27 | [-0.46, 0.97] | 78.0% | 22.0% | 7 | 25 |

## Table 2. Heterogeneity before and after adding Weekly_dose

| **Model** | **Level** | **Tau median** | **95% CrI** |
| --- | --- | --- | --- |
| Null model | Between-study | 0.17 | [0.01, 0.68] |
| Null model | Effect-level | 0.55 | [0.10, 0.98] |
| Weekly_dose model | Between-study | 0.15 | [0.01, 0.66] |
| Weekly_dose model | Effect-level | 0.53 | [0.08, 0.99] |

## Table 3. LOO model comparison

| **Model** | **ELPD-LOO** | **SE** | **LOOIC** |
| --- | --- | --- | --- |
| Null model | -39.16 | 13.92 | 78.33 |
| Weekly_dose model | -39.52 | 14.81 | 79.03 |

Difference: ELPD difference = -0.35 (SE 1.40); Delta LOOIC = 0.71.

## Table 4. Leave-one-study-out influence analysis

| **Omitted study** | **Beta** | **95% CrI** | **Pr(beta > 0)** | **Change from full beta** |
| --- | --- | --- | --- | --- |
| Goel 2023 | 0.16 | [-0.51, 0.81] | 69.1% | -0.11 |
| Jung 2019 | 0.25 | [-0.51, 0.97] | 76.1% | -0.02 |
| Kilkki 2025 | 0.19 | [-0.64, 1.01] | 67.1% | -0.09 |
| Kim 2019 | 0.18 | [-0.66, 0.97] | 67.2% | -0.09 |
| Lim 2020 | 0.26 | [-0.48, 0.98] | 75.9% | -0.02 |
| LozanoBerrio 2022 | 0.19 | [-0.64, 0.96] | 68.5% | -0.08 |
| Prasad 2018 | 0.27 | [-0.55, 1.05] | 74.3% | -0.01 |

The direction of the Weekly_dose coefficient was unchanged in all leave-one-study-out analyses. The maximum absolute change in the posterior mean beta was 0.11.

## Figure 4. Exploratory study-level Weekly_dose–effect association

Notes: Weekly_dose is expressed as digital rehabilitation minutes per week. The regression used log2-transformed Weekly_dose; therefore, beta represents the average change in Hedges' g associated with a doubling of weekly dose. Positive Hedges' g favours virtual reality or gamified digital rehabilitation. This analysis is an exploratory study-level dose–effect association and should not be interpreted as a causal dose-response relationship, an optimal dose, a threshold, or a best dose.

# 7. Leave-One-Study-Out Influence Analysis

## Table 1. Influence on the pooled SCIM effect

| **Analysis** | **Pooled SMD** | **95% CrI** | **Pr(SMD > 0)** | **CrI crosses 0** | **Change from full SMD** |
| --- | --- | --- | --- | --- | --- |
| Full model | 0.24 | [-0.12, 0.61] | 91.8% | Yes | 0.00 |
| Omit Prasad 2018 | 0.24 | [-0.23, 0.72] | 86.1% | Yes | -0.00 |
| Omit Goel 2023 | 0.18 | [-0.08, 0.45] | 92.7% | Yes | -0.06 |
| Omit Kilkki 2025 | 0.28 | [-0.20, 0.75] | 89.1% | Yes | 0.03 |
| Omit Jung 2019 | 0.25 | [-0.17, 0.66] | 90.1% | Yes | 0.01 |
| Omit Kim 2019 | 0.20 | [-0.32, 0.72] | 79.9% | Yes | -0.04 |
| Omit Lim 2020 | 0.26 | [-0.15, 0.68] | 91.2% | Yes | 0.02 |
| Omit LozanoBerrio 2022 | 0.29 | [-0.27, 0.86] | 85.7% | Yes | 0.05 |

The maximum absolute change in the pooled posterior SMD was 0.06, observed after omitting Goel 2023. The lowest P(SMD > 0) was 79.9% after omitting Kim 2019. The zero-crossing status of the 95% CrI did not change after any omission. The maximum absolute changes in between-study and effect-level tau were 0.08 and 0.46, respectively.

## Table 2. Influence on posterior heterogeneity

| **Analysis** | **Level** | **Tau median** | **95% CrI** |
| --- | --- | --- | --- |
| Full model | Between-study | 0.17 | [0.01, 0.69] |
| Omit Prasad 2018 | Between-study | 0.20 | [0.01, 0.88] |
| Omit Goel 2023 | Between-study | 0.13 | [0.01, 0.56] |
| Omit Kilkki 2025 | Between-study | 0.20 | [0.01, 0.86] |
| Omit Jung 2019 | Between-study | 0.18 | [0.01, 0.78] |
| Omit Kim 2019 | Between-study | 0.22 | [0.01, 0.94] |
| Omit Lim 2020 | Between-study | 0.18 | [0.01, 0.77] |
| Omit LozanoBerrio 2022 | Between-study | 0.24 | [0.01, 0.99] |
| Full model | Effect-level | 0.55 | [0.09, 0.99] |
| Omit Prasad 2018 | Effect-level | 0.69 | [0.25, 1.18] |
| Omit Goel 2023 | Effect-level | 0.09 | [0.00, 0.30] |
| Omit Kilkki 2025 | Effect-level | 0.70 | [0.27, 1.22] |
| Omit Jung 2019 | Effect-level | 0.59 | [0.15, 1.06] |
| Omit Kim 2019 | Effect-level | 0.78 | [0.32, 1.33] |
| Omit Lim 2020 | Effect-level | 0.59 | [0.14, 1.05] |
| Omit LozanoBerrio 2022 | Effect-level | 0.88 | [0.42, 1.48] |

## Table 3. Influence on the Weekly_dose coefficient

| **Analysis** | **Beta** | **95% CrI** | **Pr(beta > 0)** | **Change from full beta** |
| --- | --- | --- | --- | --- |
| Full Weekly_dose model | 0.27 | [-0.46, 0.97] | 78.0% | 0.00 |
| Omit Goel 2023 | 0.16 | [-0.51, 0.81] | 69.1% | -0.11 |
| Omit Jung 2019 | 0.25 | [-0.51, 0.97] | 76.1% | -0.02 |
| Omit Kilkki 2025 | 0.19 | [-0.64, 1.01] | 67.1% | -0.09 |
| Omit Kim 2019 | 0.18 | [-0.66, 0.97] | 67.2% | -0.09 |
| Omit Lim 2020 | 0.26 | [-0.48, 0.98] | 75.9% | -0.02 |
| Omit LozanoBerrio 2022 | 0.19 | [-0.64, 0.96] | 68.5% | -0.08 |
| Omit Prasad 2018 | 0.27 | [-0.55, 1.05] | 74.3% | -0.01 |

The maximum absolute change in the Weekly_dose coefficient was 0.11, observed after omitting Goel 2023. The coefficient direction remained unchanged in all omission analyses.

## Figure 5. Leave-one-study-out influence analysis

Notes: Each SCIM study was omitted once and the primary Bayesian multilevel meta-analysis was refitted. The full-model estimates are shown in blue. Panel A shows the pooled SMD and 95% credible interval. Panel B shows P(SMD > 0). Panel C shows between-study and effect-level heterogeneity. Panel D examines whether the exploratory Weekly_dose coefficient is driven by an individual study.

# 8 Prior and Model Sensitivity Analysis

## Table 1. Prior sensitivity analysis

| **Scenario** | **Studies** | **Effects** | **Pooled SMD** | **95% CrI** | **Tau_study** | **Tau_study 95% CrI** | **P(SMD > 0)** | **Status** |
| --- | --- | --- | --- | --- | --- | --- | --- | --- |
| Conservative half-normal | 7 | 25 | 0.24 | [-0.12, 0.60] | 0.16 | [0.01, 0.68] | 91.2% | Completed |
| Wider half-normal | 7 | 25 | 0.24 | [-0.12, 0.60] | 0.16 | [0.01, 0.68] | 91.2% | Completed |
| Half-Student-t | 7 | 25 | 0.24 | [-0.12, 0.60] | 0.16 | [0.01, 0.68] | 91.2% | Completed |

Across the alternative heterogeneity priors, the pooled SCIM effect ranged from 0.24 to 0.24, and P(SMD > 0) ranged from 91.2% to 91.2%. The between-study heterogeneity median ranged from 0.16 to 0.16.

## Table 2. Risk-of-bias sensitivity analysis

| **Scenario** | **Studies** | **Effects** | **Pooled SMD** | **95% CrI** | **Tau_study** | **Tau_study 95% CrI** | **P(SMD > 0)** | **Status** |
| --- | --- | --- | --- | --- | --- | --- | --- | --- |
| All studies | 7 | 25 | 0.24 | [-0.12, 0.60] | 0.16 | [0.01, 0.68] | 91.2% | Completed |
| Exclude high risk | 7 | 25 | Not estimable |  |  |  |  | Not estimable: no high-risk studies |
| Low risk / some concerns | 7 | 25 | Not estimable |  |  |  |  | Not estimable: no high-risk studies |

Risk-of-bias exclusion sensitivity was not estimable because no study was rated high risk overall. All seven studies were rated Some concerns; therefore, the all-study, exclude-high-risk, and low-risk/some-concerns datasets would be identical.

## Figure 6. Prior and model sensitivity analysis

Notes: Figure 6A compares three study-level heterogeneity priors: a conservative half-normal prior, a wider half-normal prior, and a half-Student-t prior. Figure 6B compares all studies with two risk-of-bias sensitivity scenarios. The main outcome is the pooled SCIM standardized mean difference from the Bayesian multilevel model. Posterior probabilities are not frequentist P values.

# 9: Posterior Density and MCMC Trace Plots for Model Parameters

## Table 1. Posterior summaries of principal model parameters

| **Parameter** | **Mean** | **Median** | **95% CrI** |
| --- | --- | --- | --- |
| Pooled SMD | 0.242 | 0.240 | [-0.122, 0.614] |
| Between-study heterogeneity | 0.212 | 0.165 | [0.007, 0.686] |
| Effect-level heterogeneity | 0.548 | 0.548 | [0.093, 0.994] |

## Figure 7. Bayesian model diagnostics for SCIM outcomes

Notes: Posterior histograms and chain-specific trace plots are shown for the principal model parameters. The posterior predictive density overlay compares the observed effect-size distribution with replicated datasets generated from the fitted model. The lower panels display the pooled SCIM posterior and the study-level and effect-level heterogeneity posteriors.
